# Nucleic acid-based nanotherapeutics for treating sepsis and associated organ injuries

**DOI:** 10.7150/thno.98487

**Published:** 2024-07-16

**Authors:** Huang-Ping Yu, Fu-Chao Liu, Yu-Kuo Chung, Ahmed Alalaiwe, Calvin T. Sung, Jia-You Fang

**Affiliations:** 1 Department of Anesthesiology, Chang Gung Memorial Hospital, Kweishan, Taoyuan, Taiwan.; 2 School of Medicine, College of Medicine, Chang Gung University, Kweishan, Taoyuan, Taiwan.; 3 Pharmaceutics Laboratory, Graduate Institute of Natural Products, Chang Gung University, Kweishan, Taoyuan, Taiwan.; 4 Department of Pharmaceutics, College of Pharmacy, Prince Sattam Bin Abdulaziz University, Al Kharj, Saudi Arabia.; 5 Department of Dermatology, University of California, Irvine, United States.; 6 Research Center for Food and Cosmetic Safety and Research Center for Chinese Herbal Medicine, Chang Gung University of Science and Technology, Kweishan, Taoyuan, Taiwan.

**Keywords:** sepsis, multiple organ dysfunction, bacteria, nanoparticle, nucleic acid, nucleic acid delivery

## Abstract

In recent years, gene therapy has been made possible with the success of nucleic acid drugs against sepsis and its related organ dysfunction. Therapeutics based on nucleic acids such as small interfering RNAs (siRNAs), microRNAs (miRNAs), messenger RNAs (mRNAs), and plasmid DNAs (pDNAs) guarantee to treat previously undruggable diseases. The advantage of nucleic acid-based therapy against sepsis lies in the development of nanocarriers, achieving targeted and controlled gene delivery for improved efficacy with minimal adverse effects. Entrapment into nanocarriers also ameliorates the poor cellular uptake of naked nucleic acids. In this study, we discuss the current state of the art in nanoparticles for nucleic acid delivery to treat hyperinflammation and apoptosis associated with sepsis. The optimized design of the nanoparticles through physicochemical property modification and ligand conjugation can target specific organs-such as lung, heart, kidney, and liver-to mitigate multiple sepsis-associated organ injuries. This review highlights the nanomaterials designed for fabricating the anti-sepsis nanosystems, their physicochemical characterization, the mechanisms of nucleic acid-based therapy in working against sepsis, and the potential for promoting the therapeutic efficiency of the nucleic acids. The current investigations associated with nanoparticulate nucleic acid application in sepsis management are summarized in this paper. Noteworthily, the potential application of nanotherapeutic nucleic acids allows for a novel strategy to treat sepsis. Further clinical studies are required to confirm the findings in cell- and animal-based experiments. The capability of large-scale production and reproducibility of nanoparticle products are also critical for commercialization. It is expected that numerous anti-sepsis possibilities will be investigated for nucleic acid-based nanotherapeutics in the future.

## 1. Introduction

**Figure 1:**
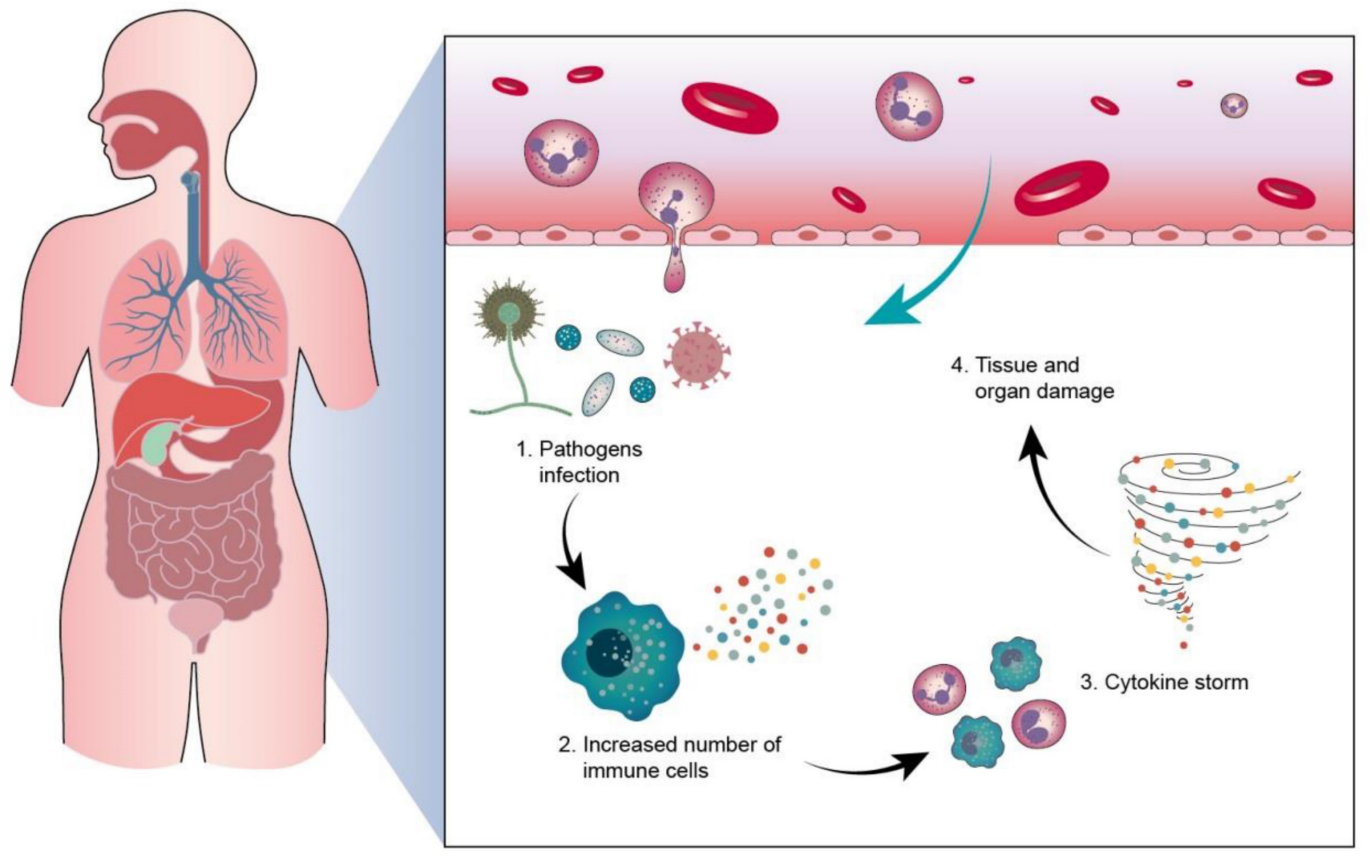
Sepsis typically originates from an infection at a specific site in the body, such as the lungs (pneumonia), kidneys (urinary tract infection), or skin (wound infection). Pathogens from the infection, such as bacteria, viruses, or fungi, enter the bloodstream and begin to spread throughout the body. The body's immune system detects the infection, initiating an inflammatory response and releasing inflammatory mediators such as cytokines. The inflammatory response may get out of control, causing the cytokine storm, leading to an excessive immune reaction and damage to normal tissues.

Sepsis is a clinical disease characterized by pathological abnormality due to pathogens, resulting in dysregulated immune response and even life-threatening organ failure. It is a leading cause of morbidity and mortality affecting all ages. Forty-nine million cases and 11 million sepsis-associated deaths were reported in 2017, accounting for about 20% of annual deaths worldwide 1. The World Health Organization (WHO) has classified sepsis as a global health priority in the next few years. Sepsis is the final common cause of death resulting from infectious diseases like viral and bacterial infections. For instance, most patients infected by Severe Acute Respiratory Syndrome Coronavirus 2 (SARS-CoV-2) experience a hyperinflammatory cytokine storm and oxidative stress damage, which are typical features of septic shock 2. In addition, sepsis-invading bacteria activate many inflammatory pathways that induce sepsis. Sepsis also can arise in other diseases where an infection triggers a systemic inflammatory reaction. These clinical conditions associated with sepsis include post-surgical infection, device-associated infection, burn, trauma, and immunocompromised state 3. Sepsis is also commonly found in the elderly population due to the age-related change in immune function and higher prevalence of comorbidities 4. The body experiences a severe response to bacterial infection characterized by bacterial existence in the bloodstream, resulting in bacteremia. Bacterial sepsis triggers an exaggerated or dysregulated inflammatory response. This hyperinflammation can be the activation of macrophages, neutrophils, and monocytes. The excessive inflammation further contributes to life-threatening organ dysfunction (Figure 1).

The physiological pathogenesis of sepsis involves a complex cascade of events triggered by an infection that leads to a dysregulated immune response and widespread inflammation throughout the body. The disease signs of sepsis include inflammatory response, microvascular dysfunction, coagulation abnormalities, organ dysfunction and failure, hypotension, and shock. Immune homeostasis maintenance and pathogen elimination are two predominant purposes for bacterial sepsis therapy. The current treatment for sepsis alleviation is the use of antibiotics, intravenous fluids for stabilizing blood pressure, vasopressors for increasing blood pressure, insulin for managing blood sugar level, and supportive care such as oxygen therapy and dialysis. However, the currently used anti-inflammatory drugs and antibiotics are usually ineffective in lowering the mortality rate 5. The major points of focus in the development of next-generation drugs for treating sepsis include targeted drug delivery, prolonged therapeutic efficacy, and reduced adverse effects. In recent years, the search for innovative therapies based on nucleic acids has emerged as highly specific anti-inflammatory or antibacterial agents for sepsis management 6. Nucleic acids can regulate the biological function of cells based on nucleotide sequencing information. Nucleic acid-based therapies-such as small interfering RNAs (siRNAs), microRNAs (miRNAs), messenger RNAs (mRNAs), long noncoding RNAs (lncRNAs), antisense oligonucleotides (ASOs), and plasmid DNAs (pDNAs)-show unique approaches to dealing with undruggable targets. This characteristic is favorable for creating a nucleic acid drug by modifying the nucleotide sequence of the target gene, contributing to the rapid and efficient development procedure 7. Nucleic acid drugs are proven to have great potential for treating viral infections, cancers, heart disorders, diabetes, and genetic diseases 8. Twenty-one nucleic acid drugs have already been approved by the United States Food and Drug Administration (USFDA) or the European Medicines Agency (Table 1).

Although nucleic acids possess outstanding features as potential drug candidates, they still face many challenges in clinical application. Naked or unmodified nucleic acids often have unfavorable physicochemical properties, including anionic charge, large molecular size, and instability in physiological conditions 9. These limitations result in poor permeability into the cells, off-target effect, and quick elimination by serum nucleases *in vivo* 7. Nucleic acids also induce immunogenicity, raising concerns about toxicity 10. Some strategies, such as N-acetylgalactosamine conjugation, polymeric conjugation, viral vectors, and engineered nanocarriers, are employed to resolve the drawbacks of nucleic acid drugs 11. Among these, the non-viral nanocarriers for nucleic acid-based drug delivery exhibit an important capacity in improving cellular uptake, instability, and immunogenicity. Nucleic acid nanocarriers can be designed to target specific inflammatory mediators or pathways involved in sepsis. This targeted approach aims to reduce systemic inflammation and prevent organ damage. Preclinical studies have demonstrated that nucleic acid-based therapies delivered via nanocarriers can modulate immune responses, improve bacterial clearance, and enhance survival in animal models of sepsis 12. Various types of nanocarriers, such as liposomes, polymeric nanoparticles, and lipid nanoparticles, are explored for delivering nucleic acids. Each type has its advantages and considerations regarding stability, biocompatibility, and target specificity. The search results indicate that advanced nanoscale biotechnology has enabled the development of various nucleic acid nanocarriers with promising applications for sepsis diagnosis and treatment 13. However, further clinical evaluation and optimization are required before these nanotechnology-based approaches can be widely adopted.

The successful outcomes for clinical sepsis management should be timely diagnosis and early and effective therapeutic intervention. Over a hundred clinical trials are conducted for therapeutic treatment related to sepsis. However, no standard treatment option has been approved by the USFDA until to this day 14. The exploration of novel and effective therapies to alleviate the symptoms of the increase in the survival rate of sepsis is critical. This review summarizes the opportunities for nanoparticle use for nucleic acid-based therapeutics to treat sepsis and related organ injuries. In this paper, a section is dedicated to presenting various types of nucleic acids employed for treating sepsis. The different nanoparticle types applied for nucleic acid-based nanotherapeutics are also introduced in the text. Finally, an exhaustive review of the previous investigations involved in sepsis treatment by nucleic acid-loaded nanocarriers is described.

## 2. Nucleic acids used for treating septic inflammation

**Figure 2:**
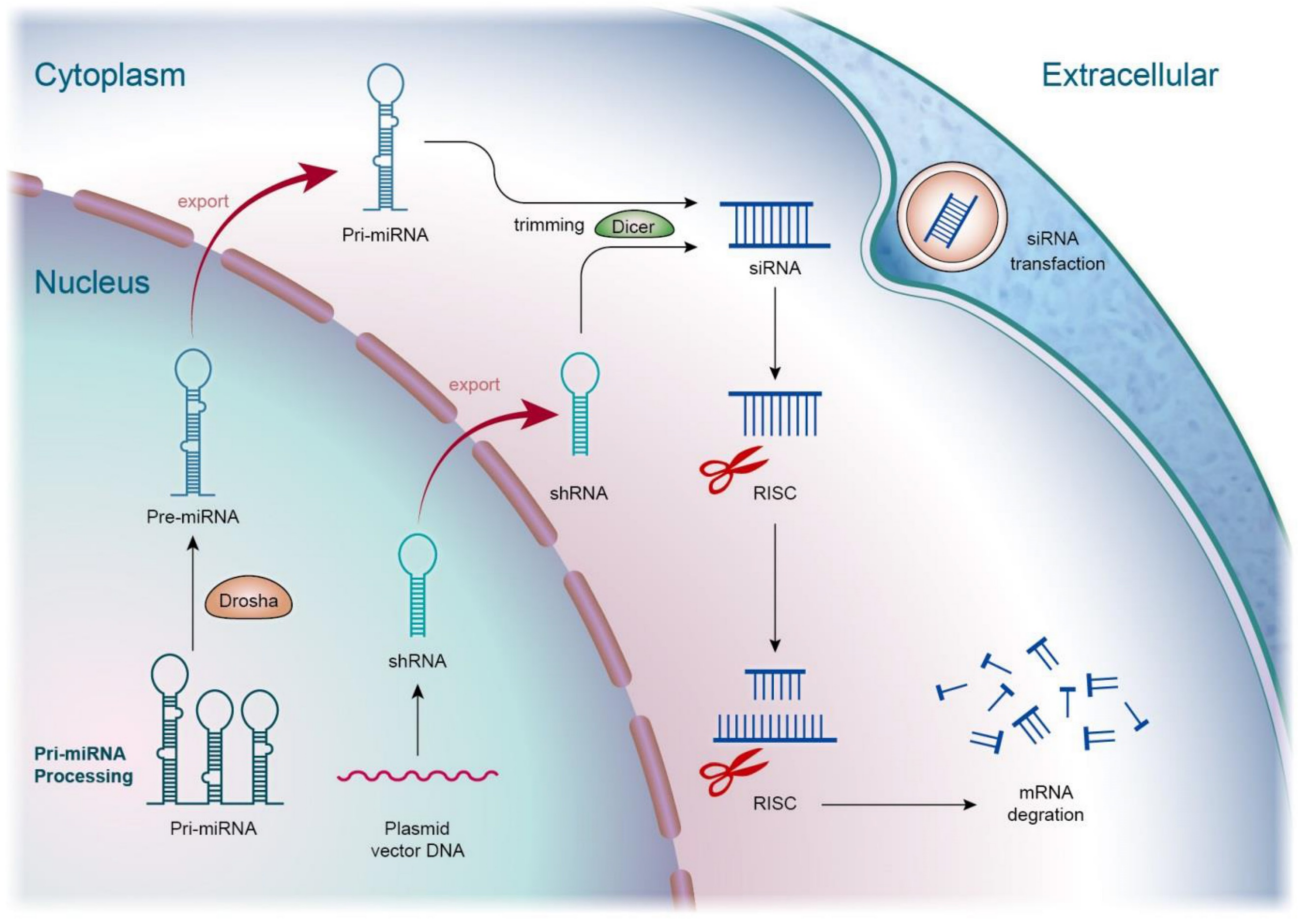
The mechanism of siRNA involves several key steps in the RNA interference (RNAi) pathway, leading to the specific suppression of gene expression. Exogenous or endogenously produced siRNA is processed into short double-stranded molecules, typically by enzymes like Dicer within cells. These siRNA molecules then bind to the RNA-Induced Silencing Complex (RISC), forming an active RISC complex. Through complementary base pairing, one strand of the siRNA molecule guides RISC in identifying and binding to the target mRNA. This process effectively regulates gene expression, making siRNA a powerful tool in biological research and a potential therapeutic approach for various diseases.

Inflammation inhibition to prevent the hyperactivation of cells and tissues is a crucial strategy to alleviate the symptoms of sepsis. Though the inflammatory response is a protective manipulation to neutralize the pathogens, toxins, and irritants, inappropriate inflammation creates tissue damage. Bacteria can stimulate toll-like receptors (TLRs) of macrophages, mast cells, and dendritic cells to activate excessive inflammation 15. This stimulation elicits the production of cytokines and chemokines, possibly inducing sepsis. Apart from the role of regulating the bioprocesses of the cells, optimal nucleic acids can exhibit the inflammation inhibitor through the suppression of proinflammatory mediators or the elevation of anti-inflammatory mediators for the return to homeostasis. This effect of nucleic acids is valuable in treating inflammatory and autoimmune disorders. Among the different types of nucleic acids, both siRNAs and miRNAs are largely used for treating sepsis through anti-inflammatory mechanisms. siRNA is a nucleic acid drug for inhibiting inflammation caused by sepsis, as well as subsequent organ injury. This nucleic acid is a double-stranded RNA consisting of 20‒30 nucleotides. It is a class of RNA inhibitors acting by RNA-induced silencing complex (RISC) that specifically degrade target RNAs in cells 16. RNA interference (RNAi) involves the introduction of dsRNA into the host cells to prompt post-transcriptional silencing of homologous host genes and transgenes. The RNAi process starts with the enzyme breaking down dsRNA into smaller RNA duplexes (siRNA). Then, siRNA is incorporated into RISC. The endonuclease argonaute 2 (Ago-2) component of RISC unwinds the duplex into two single-stranded RNAs. This process leads to the admission of the antisense strand, binding to the target RNA molecules through complex RISC 17. Finally, the complex cleaves the target mRNA mediated by Ago-2, resulting in mRNA translation inhibition and target gene knockdown (Figure 2).

**Figure 3:**
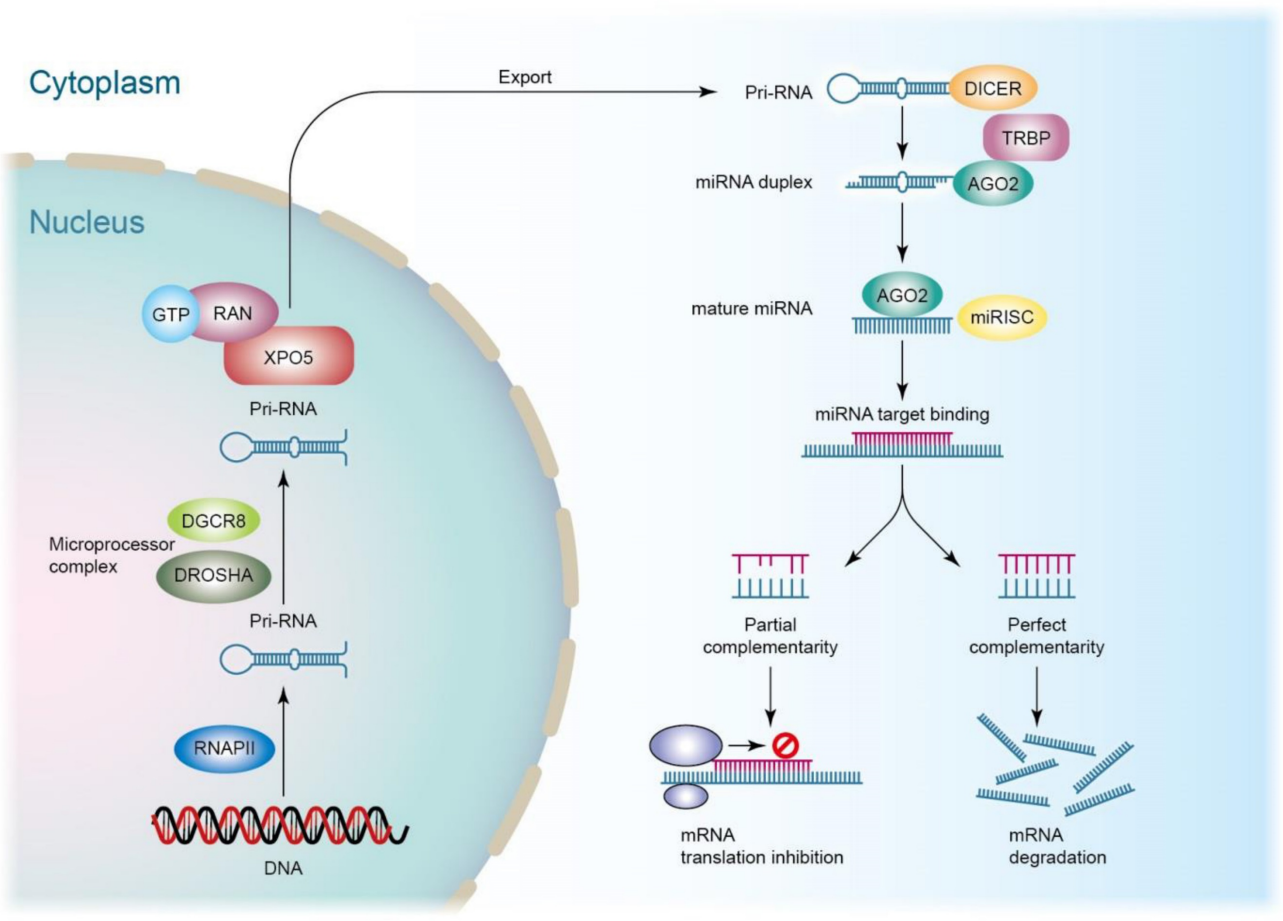
miRNA (microRNA) are short, non-coding RNA molecules, typically composed of about 20 to 25 nucleotides. They play crucial roles in gene expression regulation, capable of inhibiting the expression of target genes. It is transcribed from cell genes, forming long primary miRNA (pri-miRNA) transcripts. These long precursor miRNAs undergo processing by a series of nucleases, including Drosha and Dicer, producing mature miRNA molecules. Mature miRNA binds to the RNA-Induced Silencing Complex (RISC), forming an active miRNA-RISC complex. This complex pairs with the 3' untranslated region or other regions of target mRNA through partial complementarity, leading to degradation or translational repression of the target mRNA. miRNA plays critical roles in regulating gene expression, influencing various biological processes such as cell proliferation, differentiation, apoptosis, and development. A single miRNA may regulate the expression of multiple genes. If there is complete complementarity, it leads to RISC guiding the degradation of the target mRNA. If there is partial complementarity, it inhibits the translation of the target mRNA, thereby reducing the protein output of the gene.

siRNAs are advantageous for therapeutic application because of their easy synthesis, minimized risks of mutation and teratogenicity, low immune response, and satisfied specificity and potency 18. Recruitment and activation of macrophages and other immune cells is a consequence of septic inflammation. Cytokines/chemokines can be potential targets for anti-sepsis therapy. Prime candidates, such as interleukin (IL)-20, IL-23, and tumor necrosis factor (TNF)-α-encoding mRNAs, are recognized as specific targets for RNAi 19. Compared to siRNAs, miRNAs show stronger inflammation inhibition since they affect multiple cellular processes rather than specific targets because miRNA recognition needs binding to shorter seed sequences instead of the entire nucleotide sequence of siRNAs 20. One miRNA can modulate several genes that usually act in the same biological pathway. miRNAs are small and highly conserved transcripts containing 19‒25 nucleotides. The single-stranded RNAs regulate gene expression through the induction of mRNA degradation. miRNAs are transcribed from deoxyribonucleic acid (DNA) sequences in the nucleus by RNA polymerases 21. Drosha is a member of the RNase III family, cleaving the primary miRNA (pri-miRNA) to generate a 70-nucleotide precursor miRNA. The precursor miRNA transports to the cytoplasm by exportin-5, which is then processed by the RNase III endonuclease dicer to produce mature miRNA. Subsequently, the mature miRNA is loaded onto RISC as guided by the Ago family to bind to the 3′-untranslated region (3′UTR) of the target mRNAs 22. This mechanism can result in the translation suppression or degradation of the target mRNAs (Figure 3). Exosomes can protect miRNAs and neglect the enzymatic degradation *in vivo*. The exosomes with abundant miRNAs are valuable as nanocarriers for treating septic inflammation.

miRNAs are reported to be involved in the regulation of inflammation, metabolic pathways, differentiation, and apoptosis of the cells 23. Two therapeutic strategies are used for miRNA-based regulation: miRNA antagomirs (inhibitors) and agomirs (mimics). The antagomirs are complementary to the target miRNAs and act as antagonists via the blockade of the binding to endogenous mRNAs. On the other hand, agomirs are chemically designed to simulate endogenous miRNAs for restoring miRNA levels that are reduced in the diseases 24. miRNAs act as a key regulator, controlling inflammation in sepsis 25. Some anti-inflammatory miRNA agomirs are reported to mitigate inflammation 26, including miR-10a, miR-21, miR-24, miR-106b, miR-124, miR-143, miR-145, miR-146, miR-155, and miR-375. These mimics act as inflammation regulators by targeting inflammation pathways, such as TLRs, nuclear factor-κB (NF-κB), Janus kinase (JAK), and TNF receptor-associated factor 6 (TRAF6). The increased level of these mimics in the cells or tissues abrogates the release of proinflammatory cytokines/chemokines, attenuating the inflammatory reaction 27.

**Figure 4:**
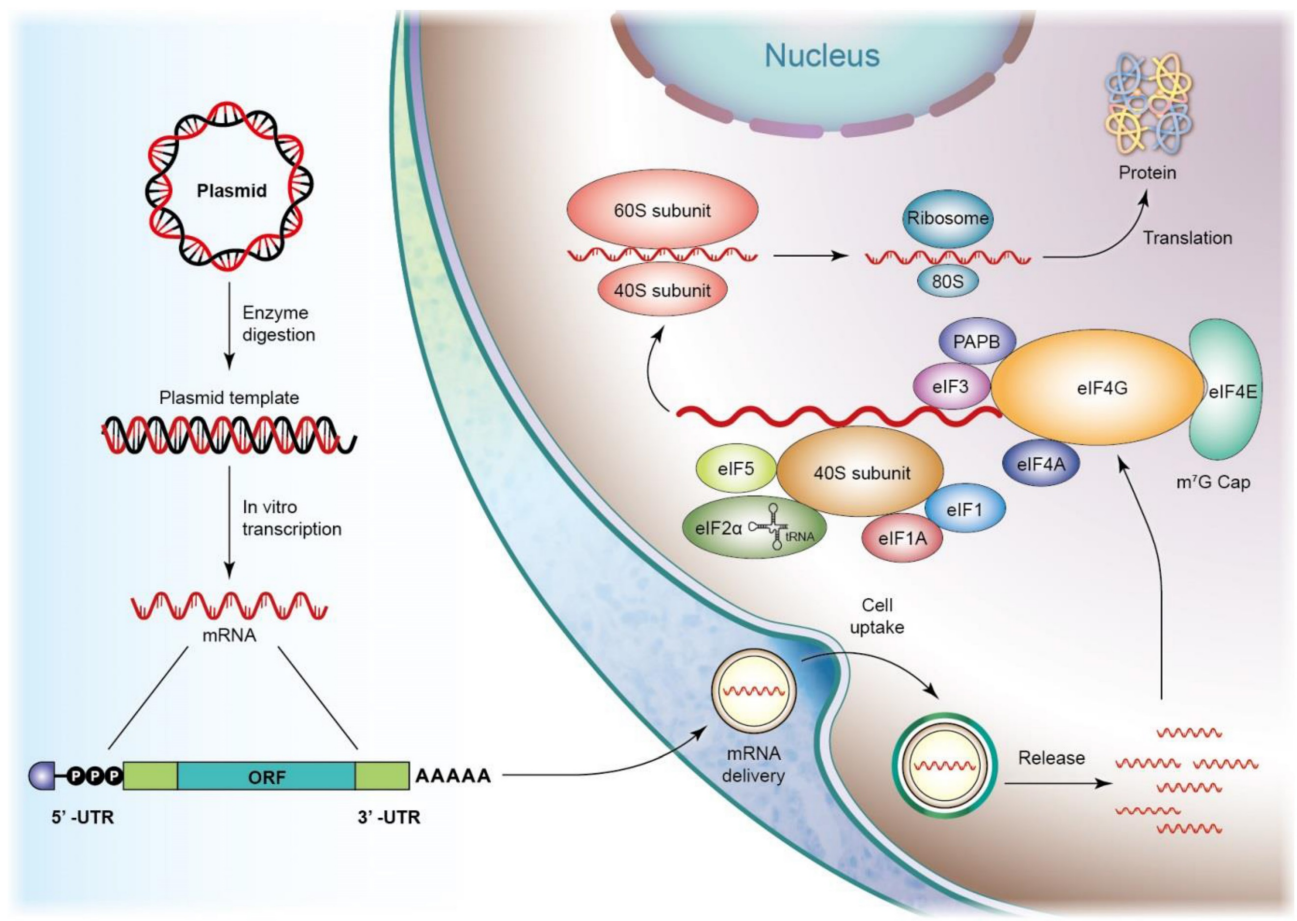
*In vitro* transcribed (IVT) mRNA and translation initiation. IVT mRNA preparation includes several steps: plasmid cloning, plasmid linearization, *in vitro* transcription, 5' capping, and the poly(A) tail adding. After mRNA enters the cell, mRNA translation may commence through an elF4F-dependent mechanism, promoting the recruitment of a preinitiation complex. (PIC). The 43S PIC is shaped by the 40S ribosomal subunit, the eukaryotic translation initiation factors (elF, containing elF1, elF1A, elF3, elF5), and the ternary complex, containing a trimeric complex comprising elF2 that contains α-, β-, and γ-subunits, initiating methionyl tRNA, and GTP. elF4F is a complex composed of elF4A, elF4E and elF4G. The mRNA cap is bound by elF4E. elF4G interacts with elF3 and poly(A)-binding protein (PABP) that binds to the 3' poly(A) tail. These interactions result in mRNA circularization and assembly of the 48S PIC. The 48S PIC ribosomal subunit scans and finds the start codon with the help of elF4A helicase to resolve the secondary mRNA structure in the 5' UTR. Finally, elFs are released, and the 60S ribosomal subunit joins to initiate translation elongation by forming the 80S ribosome to produce the aim protein.

Sepsis-associated inflammation and organ failure are also mitigated using mRNA drugs. mRNAs are single-stranded ribonucleic acids composed of hundreds to thousands of nucleotides that are translated to corresponding proteins through protein synthesis machinery. *In vitro*-transcribed (IVT) mRNAs are structurally similar to the processed mRNAs in cells, offering genetic information that enables the translational machinery of the host cells to produce functional proteins 28. After penetrating the cells, IVT mRNA translation starts in an eIF4F-dependent pathway to recruit a preinitiation complex (PIC). The 43S PIC is formed by the eukaryotic translation initiation factors (eIF) and the ternary complex, including a trimeric complex comprising eIF2 that contains α-, β-, and γ-sub-units. eIF4F is composed of eIF4A, eIF4E, and eIF4G. eIF4E binds to mRNA, and eIF4G interacts with eIF3 and poly(A)-binding protein (PABP) that binds to the 3′ poly(A) tail. These interactions result in mRNA circularization and 48S PIC assembly 29. The 48S PIC ribosomal sub-unit scans the start codon with the assistance of eIF4A helicase to resolve the secondary mRNA structure in the 5′ UTR. Finally, eIFs are released, and 60S ribosomal sub-unit joins in initiating translation elongation by forming an 80S ribosome (Figure 4). mRNAs initiate protein translation after reaching the cytosol without the need to deliver into the nucleus, minimizing the toxic risk of inserting into the genome 30. Further, mRNAs are easily synthesized via the IVT approach, promising fast and predictable protein expression kinetics. The therapeutic application of mRNAs includes vaccines, protein replacement, infectious disease management, cancer immunotherapy, and cellular reprogramming. The mRNA vaccines formulated in lipid nanoparticles are approved for emergency use against SARS-CoV-2 31. lncRNAs and polyadenylated transcripts containing >200 nucleotides are spliced, resulting in their description as mRNA-like. The difference between lncRNAs and mRNAs is the localization of a greater amount of lncRNAs in the nucleus 32. lncRNAs mediate epigenetic modification via the recruitment of chromatin-remodeling complex to the specific chromatin locus 33. However, the detailed mechanisms of how specific a DNA region is targeted by lncRNAs need further elucidation. Some evidence has shown that lncRNAs can act as therapeutic targets of sepsis. lncRNA-HOTAIR enhances the production of TNF-α in LPS-induced sepsis in mice 34. lncRNA-MALAT1 accelerates skeletal muscle cell apoptosis and inflammation in sepsis 35.

Both ASO and pDNA are DNA molecules. ASOs are short single-stranded DNA, ranging from 13‒30 bases in length. miRNAs and mRNAs can be targeted by ASOs, which leads to the suppression of gene function. ASOs can selectively inhibit disease-associated genes by ribonuclease H-mediated cleavage or steric hindrance 36. In addition to the elimination of protein expression, ASOs also enhance target translation. The drawback of the clinical application of ASOs lies in their low affinity with plasma proteins, resulting in quick elimination from the bloodstream after intravenous (IV) administration 37. Moreover, ASOs are rapidly digested by intracellular endo- or exonucleases. For increasing the stability, nanoparticle inclusion can resolve the challenges of delivery. pDNAs are small circular DNA molecules that can be genetically engineered to carry therapeutic genes into cells. The most used DNA-based vectors for gene therapy and DNA vaccination are plasmids 38. pDNA for gene therapy and DNA vaccination offer several advantages over other nucleic acids, such as being easy to design and manufacture, having a low cost, and having satisfied stability for long-term storage. The nucleic acids mentioned in this section have been applied as bioactive agents inside the nanoparticles for treating sepsis. On the other hand, some nucleic acids, such as pDNAs, are utilized as materials in anti-sepsis nanoparticles for improving stability and delivery efficiency.

## 3. The types of nanocarriers used for nucleic acid-based nanotherapeutics

**Figure 5:**
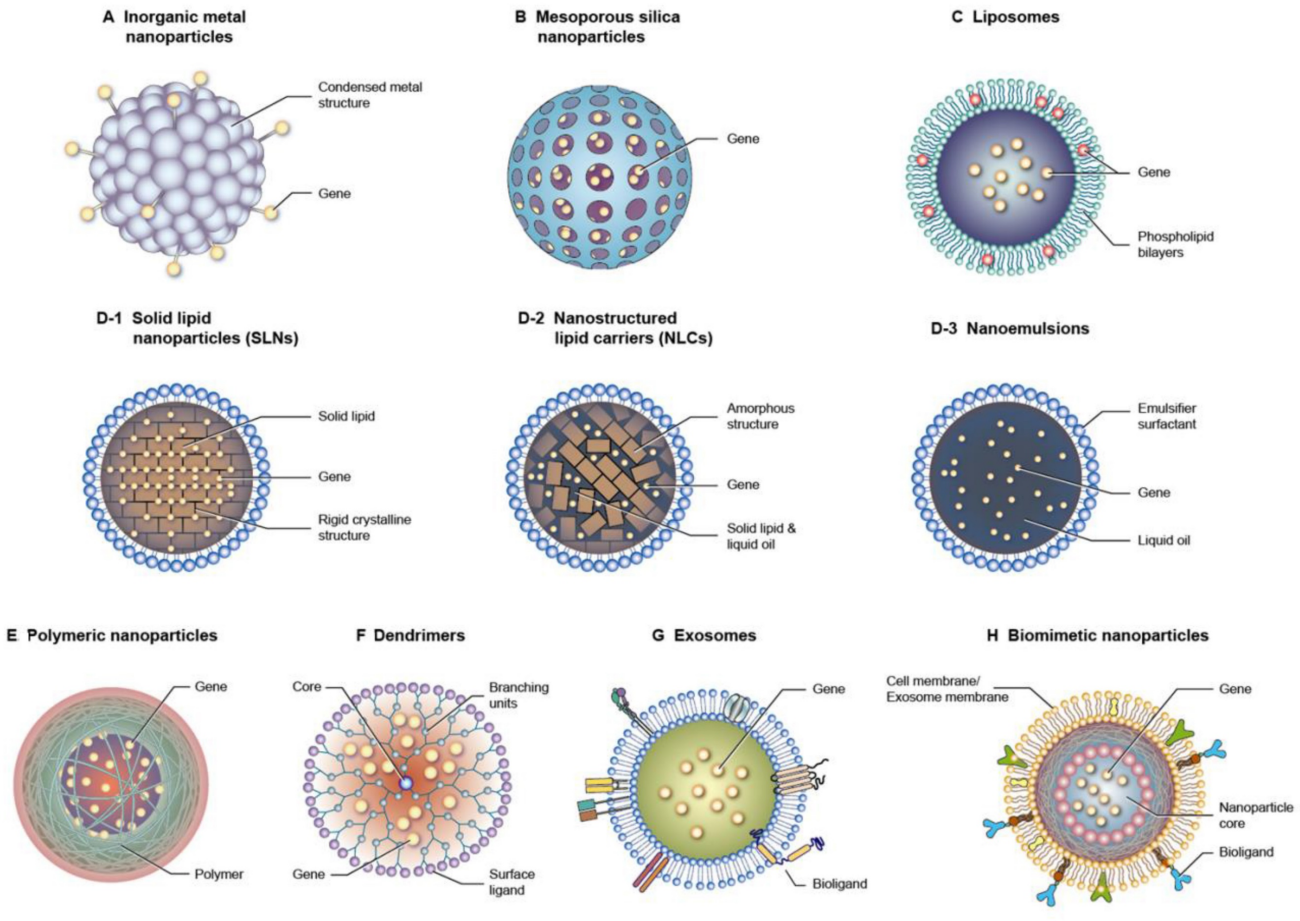
Different types of nanocarriers used for gene delivery. (A) Inorganic metal nanoparticles (B) Mesoporous silica nanoparticles (C) Liposomes (D-1) Solid lipid nanoparticles (SLNs) (D-2) Nanostructured lipid carriers (NLCs) (D-3) Nanoemulsions (E) Polymeric nanoparticles (F) Dendrimers (G) Exosomes (H) Biomimetic nanoparticles.

One of the most important factors for successful therapy using nucleic acid drugs is the efficient delivery to target tissues and cells. Nucleic acid-based molecules have difficulty penetrating across the cell membrane and tissue barrier because of their large size, high hydrophilicity, and polyvalent anionic characteristics. The instability, rapid clearance, and immunogenicity of nucleic acids also cause obstacles to their clinical application 39. Nucleic acid delivery by nanocarriers has a mechanism that overcomes the challenges associated with targeting. Gene therapy with the nanoparticle delivery system has elevated the biomedical application of the treatment of sepsis and subsequent organ dysfunctions. The nanoparticles are designed from inorganic or organic materials with specific physical, chemical, and surface features to exhibit the desired biological function or properties. The nanocarriers show the advantages of nucleic acid delivery such as protection from biodegradation, enhanced cell uptake, targeted delivery, controlled release, biocompatibility, and versatility in payloads 40. While the nanoparticles provide benefits for nucleic acid delivery, they also present some challenges including complexity of design and production, toxicity, non-specific uptake, immune activation, batch-to-batch variability, and long-term instability 41. Nanoparticles can encapsulate nucleic acids by entrapment, adsorption, and covalent binding 42. At the cell level, the nanoparticles provide an efficient approach to promote cellular uptake and lysosomal escape, which is beneficial to accomplish therapeutic aims, such as gene silencing, protein replacement, and vaccination. Several types of nanoparticles are utilized for gene delivery with the goal of optimizing nucleic acid therapy against sepsis (Figure 5).

### 3.1. Metal-based inorganic nanoparticles

Some inorganic metals, such as gold, silver, titanium, and iron, are used as nanomaterials for nucleic acid delivery because of their tiny and controllable size and shape. Since the metallic nanoparticles are very small (generally <20 nm), it is difficult to include the nucleic acids in the core. Most of the cases of nanoparticle loading include the covalent attachment of nucleic acid strands with the nanoparticulate surface via thiol moieties or electrostatic interaction 43. The thiol-conjugated surface provides a stable condition outside of cells but is degraded intracellularly due to the high glutathione concentration inside the cells 44. It is reported that some inorganic metal nanoparticles can restrain the immune response 45. This biological property of metal nanoparticles is expected to be beneficial for synergistically alleviating septic inflammation.

### 3.2. Mesoporous silica nanoparticles (MSNs)

MSNs feature a nanoparticle form with a porous network in the silicon oxide matrix or on the nanoparticulate surface. The unique physicochemical properties of MSNs, such as large surface area, tunable particle size and porosity, and biocompatibility, are ideal carriers for drug delivery 46. The therapeutic use of MSNs is prominent due to their excellent performance in delivering nucleic acids, including siRNAs and mRNAs, at target sites. Nucleic acids are generally loaded into MSNs via weak, non-covalent interaction 45. The functionalization with cationic components is usually required to achieve a high loading of nucleic acids 47. The pore size of MSNs can be optimized to fabricate the ideal carriers for nucleic acid delivery.

### 3.3. Liposomes

Liposomes are lipid nanovesicles composed of phospholipid bilayers with an aqueous inner core. Stabilizers such as cholesterol are needed to stabilize the bilayer structure. The structure of phospholipid bilayers simulates the cell membrane, resulting in the preferable interaction and facile fusion with the cells. Another advantage of liposomes is their biodegradability and biocompatibility, which verifies their safe use in clinical applications 48. Cationic lipids like 1,2-dioleoyl-sn-glycero-3-phosphoethanolamine (DOPE), 1,2-dioleoyl-3-trimethylammonium propane (DOTAP), and *N*-[1-(2,3-dioleyloxy)propyl]-*N*,*N*,*N*-trimethylammonium (DOTMA) are incorporated into liposomes to exploit electrostatic attraction with nucleic acids 45.

### 3.4. Lipid-based nanoparticles

Unlike liposomes, lipid-based nanoparticles have a lipid core coated by single-layered surfactants or emulsifiers. They generally disperse in an aqueous environment as the external phase. Lipid nanoparticles are nanosized and have improved lipid solubility, leading to high drug encapsulation and increased bioavailability. The scale-up for industrial production is possible for lipid-based nanoparticles, elevating the applicability in biomedical and clinical uses 49. According to the lipid core structure, lipid-based nanoparticles are classified as nanoemulsions, solid lipid nanoparticles (SLNs), and nanostructured lipid carriers (NLCs) 50. The difference between the three types of lipid nanoparticles predominantly depends on the composite of the lipid cores. Lipid-based nanocarriers are one of the most broadly studied vehicles for nucleic acid delivery 51. Similar to liposomes, cationic lipids must be incorporated into lipid-based nanoparticles to achieve an efficient encapsulation of nucleic acids 52. The addition of helper lipids such as phospholipids and cholesterol - the major ingredients of liposomes - can facilitate the stability and cell membrane fusion for valid nucleic acid targeting. Polyethylene glycol (PEG) lipid decoration on the surface of lipid nanoparticles provides steric hindrance from enzymatic degradation in the bloodstream, improving the half-life and stability of nucleic acid drugs 53. The lipid-based nanoparticles can conjugate with ligands, such as antibodies, aptamers, and peptides, to recognize and bind to specific cell receptors. A remarkable success of lipid nanoparticles is found in delivering siRNAs and mRNAs as rare inherited disease treatments and SARS-CoV-2 vaccines, respectively 54.

### 3.5. Polymer-based nanoparticles

Polymer-based nanosystems are colloidal nanoparticles consisting of macromolecular materials derived from synthetic or natural resources. The synthetic polymers frequently applied for preparing polymeric nanoparticles include poly(lactic-*co*-glycolic acid) (PLGA), polylactic acid (PLA), PEG, polyamidoamine, poly(ε-caprolactone), ethylcellulose, cellulose acetate phthalate, poly(butyl cyanoacrylate), and poly(vinyl alcohol). Dextran, chitosan, and hyaluronic acid (HA) are the commonly used natural polymers for producing polymeric nanoparticles. Polymer-based nanoparticles are promising vehicles for nucleic acid delivery owing to their easy synthesis and functionalization, structure versatility, scalability, high transfection efficacy, and good biocompatibility 55. Polyethyleneimine (PEI) and poly-L-lysine are considered polycations to increase nucleic acid encapsulation 45. The positively charged surface also strengthens the cellular uptake of the polymeric nanoparticles. Chitosan, with its cationic nature, is ideal for obtaining a high nucleic acid encapsulation without the need for polycations 56. Noteworthily, nucleic acids are biopolymers. Besides the role of bioactive agents in the polymer nanoparticles, nucleic acids can serve as materials for fabricating the nanoparticles. The unique properties of nucleic acids - such as their ability to form specific and programmable structures through base pairing - make them attractive for designing nanoparticles with precise control over size, shape, and functionality 57.

### 3.6. Exosomes

**Figure 6:**
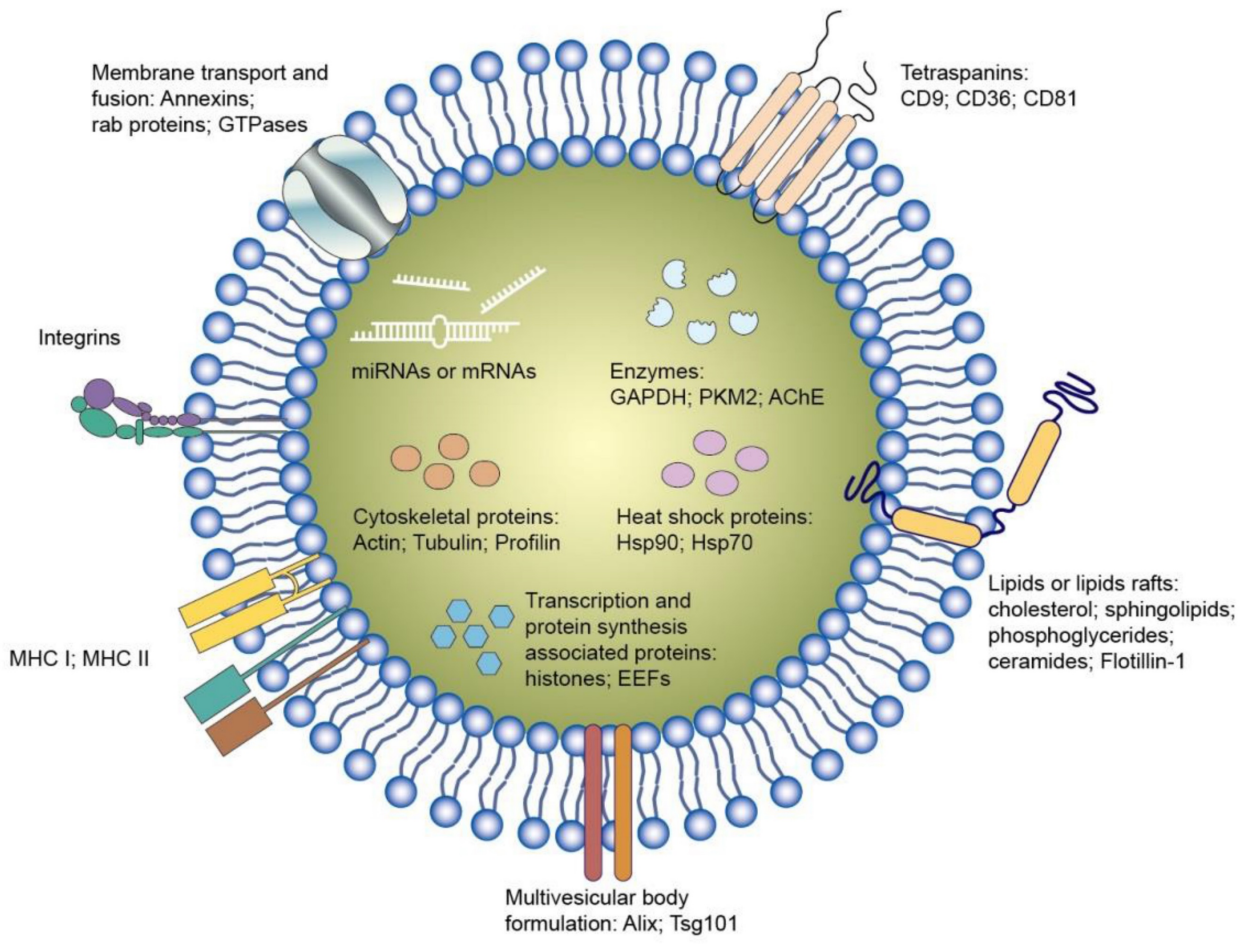
Exosomes are small membrane-bound vesicles that are secreted by various cells throughout the body. They play a crucial role in intercellular communication by carrying proteins, enzymes, lipids, RNA, and other molecules between cells. Research into exosomes has expanded rapidly in recent years, driven by their potential applications in diagnostics, therapeutics, and drug delivery. Their ability to transport bioactive molecules to target cells has made them promising candidates for delivering therapeutic agents, such as drugs, nucleic acids, and proteins, with high specificity and minimal side effects.

Exosomes are endogenous nanoparticles consisting of lipid bilayers. It is thought that exosomes or EVs are responsible for intercellular communication. Several nucleic acids, proteins, and lipids inside the exosomes play a vital role in regulating or modifying the bioprocesses of the recipient cells (Figure 6). Exosomes can facilely pass through tissue barrier and cell membranes with low immunogenicity. They also present the advantages of compatibility with living tissues, low toxicity, extended blood circulation, and special targeting of specific cells, making them robust therapeutic nanocarriers 58. It is also possible to use exosomes as delivery systems for synthetic nucleic acids 59. The nucleic acids can be entrapped into the isolated exosomes from the cells. Some factors restrict the therapeutic application of cell-derived exosomes, such as barriers related to exosome isolation, characterization, quality check, fast circulation clearance, and unsatisfactory targeting capability 60. The engineered exosomes can be employed as an effective approach to overcome the limitations and expand their loading capacity for nucleic acids 61. Incorporating specific ligands can enhance the cell targeting of the exosomes. Exosome stability is also promoted via exosome engineering by means of physical or chemical treatment such as PEGylation. Targeted nucleic acid delivery can also be achieved by exosome manipulation through magnetic stimuli 62. The potential therapeutic activity of exosomes against sepsis and bacterial inflammation has been explored in the last decade. Its major mechanism involves the selective delivery of cellular cargos, such as miRNAs and mRNAs.

### 3.7. Biomimetic nanoparticles

The challenges of cell-derived exosomes for delivery systems have led to the engineering of biomimetic nanoparticles. Biomimetic nanosystems are bioinspired nanoparticles based on coating the surface of synthetic nanoparticles with a natural cell membrane or exosome membrane. This type of nanosystem retains the physicochemical characteristics of synthetic nanomaterials and inherits the specific nature of cellular or exosome membranes. Biocompatibility, enhanced biointerfacing capability, cellular function maintenance, immunological escape, and prolonged circulation half-life are observed for biomimetic nanoparticles 63. Over the years, SARS-CoV-2, cancers, and atherosclerosis have been mainly investigated to confirm the effectiveness of biomimetic nanoparticles. The application of biomimetic nanoparticles for alleviating the symptoms of sepsis has been recently evaluated 14. Some natural mechanistic pathways of the immune response related to sepsis are simulated by the biomimetic nanoparticles to achieve sepsis microenvironment targeting. The synthetic nanoparticles can be decorated by the membranes of immune cells, erythrocytes, platelets, and EVs from leukocytes for effective treatment of sepsis and the associated organ damage 64.

**Figure 7:**
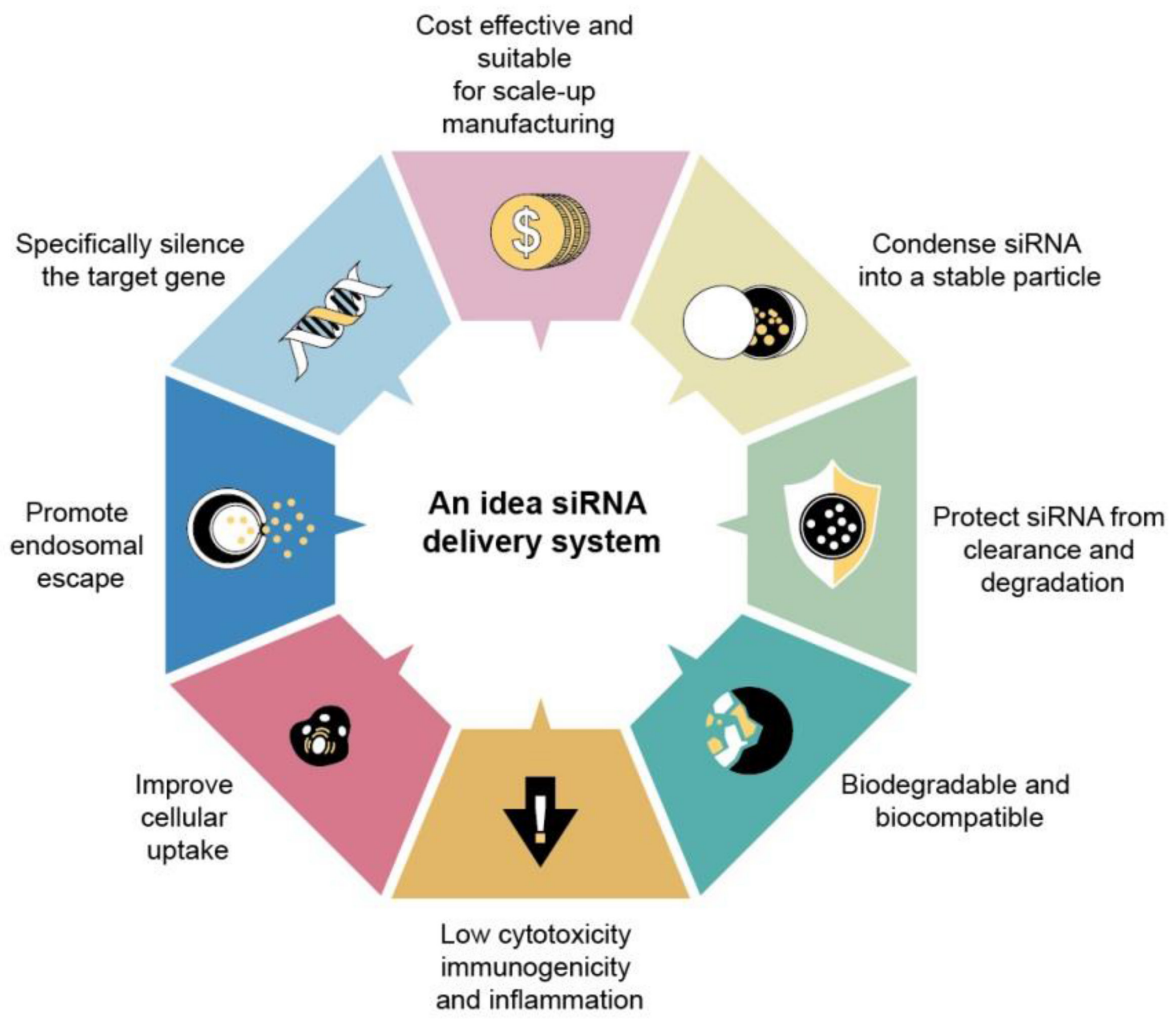
An ideal siRNA delivery system should have high efficiency, specificity, safety, stability, targeting ability, controllability, and ease of preparation. This entails the effective and stable delivery of siRNA to target cells, enabling targeted action. Moreover, the system should ensure safety by avoiding toxicity or immune reactions and maintaining stability *in vivo*. Additionally, it should be easy to prepare, with simple, cost-effective, and scalable production methods to meet clinical application requirements.

Notably, nucleic acid-loaded nanocarriers still face challenges, particularly low encapsulation efficiency, unsatisfied stability, and off-targeting. IV injection is the administration route for most of the anti-sepsis nanoparticles. Prolonged retention in circulation is also important to extend therapeutic efficacy. For possible human applications, the ideal nanodelivery systems for nucleic acids should include the following capabilities (Figure 7): (i) high entrapment into stable nanoparticles, (ii) protection of nucleic acids from enzymatic attack, (iii) the high transfection efficiency to facilitate cellular ingestion, and (iv) the facile lysosomal escape and cytosolic release of nucleic acids. It is recommended that the nanocarriers be biocompatible without immunogenicity and toxicity for safety concerns.

## 4. Sepsis and the associated organ failure

**Figure 8:**
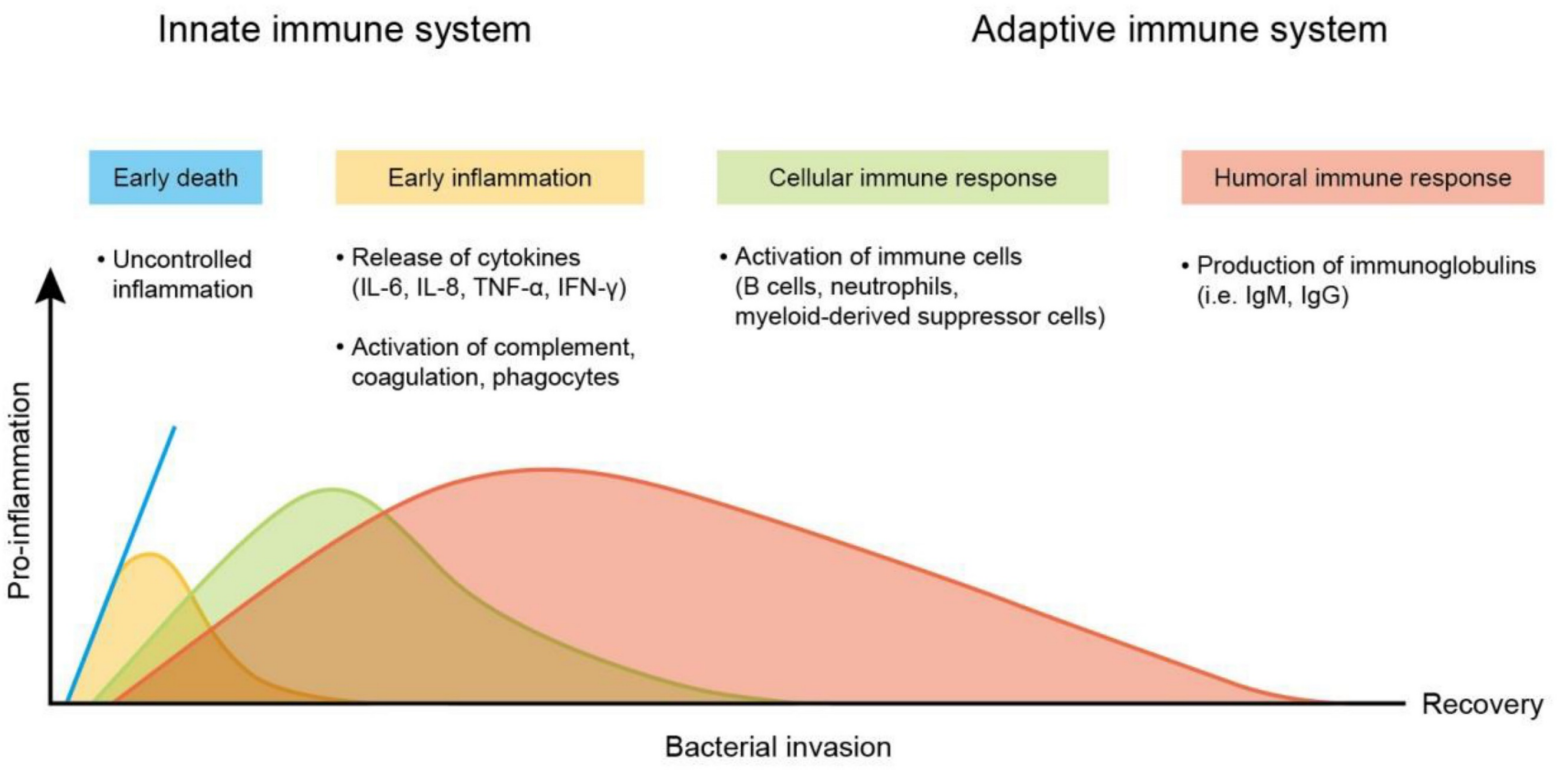
Changes in pro-inflammatory response of the immune system during the course of sepsis. The peak mortality rates during the early period (blue line) were due to overwhelming inflammatory response, called “cytokine storm,”. It can cause comprises fever, refractory shock, inadequate resuscitation, and cardiac or pulmonary failure. The mortality at the later period is due to persistent immunosuppression with secondary infections that results in organ injury and failure.

Sepsis is a life-threatening disease mainly caused by systemic bacterial infection and triggering hyperinflammatory reactions. The two predominant pathogenesis factors of sepsis are invading bacteria and host immune response. The most common bacteria responsible for sepsis include *Staphylococcus aureus*, *Escherichia coli*, *Pseudomonas* species, and *Klebsiella* species. The main clinical problem of bacterial infection is acquired antibiotic resistance - a threat to public health 65. Antibacterial resistance has become a challenge for the therapeutic treatment of sepsis. *S. aureus*, *Streptococcus pneumonia*, *Acinetobacter*, *Enterobacter*, and *Haemophilus* appear as deadly multidrug-resistant bacteria 66. After infection in the bloodstream, bacteria survive oxidation on the erythrocyte surface and enter into erythrocytes for proliferation. The proliferated bacteria largely produce toxins. These toxins serve as virulence factors that interact with the immune cell membrane via the targeting of proteins and lipids 67. They can also bind to the membrane via nonspecific electrostatic attraction. Lipopolysaccharide (LPS) in the outer membrane of most Gram-negative bacteria is endotoxin, a virulence factor that results in sepsis. The toxins activate the immune cells to express a high level of pro-inflammatory mediators, referred to as cytokine storm 68. The cytokine storm evokes an overwhelming inflammation, leading to high fever and refractory shock, followed by peripheral organ failure (Figure 8). In addition, cytokine storms may be responsible for the high death rate among patients. The late stage of sepsis is the exhaustion of the immune system, immune cell dysfunction, and apoptosis, contributing to immunosuppression and late-period mortality 69. Sepsis and the related cytokine storm affect different functions and sites in the body, including circulation and peripheral organs. These complications are always fatal. To increase the survival rate of sepsis patients, it is important to treat the symptoms in circulation and peripheral organs.

### 4.1. Bacteremia

For diagnosing sepsis, the blood sample is collected for culturing bacteria. Bacteremia is the presence of invading bacteria in the bloodstream. It can occur among sepsis patients. Bacteremia triggers acute inflammation in circulation, leading to sepsis and multiple organ damage 70.* S. aureus* bacteremia-induced sepsis is a major cause of bloodstream infection, with a mortality rate of ~25% 71. Notably, the death rate from Methicillin-resistant *S. aureus* (MRSA) bacteremia is even higher. IV antibiotics are delivered in patients with bacteremia after appropriate cultures of potential sources and blood are obtained. Advanced treatment of bacteremia with an appropriate antibacterial regimen is vital to improve the survival rate of patients.

### 4.2. Acute lung injury (ALuI)

**Figure 9:**
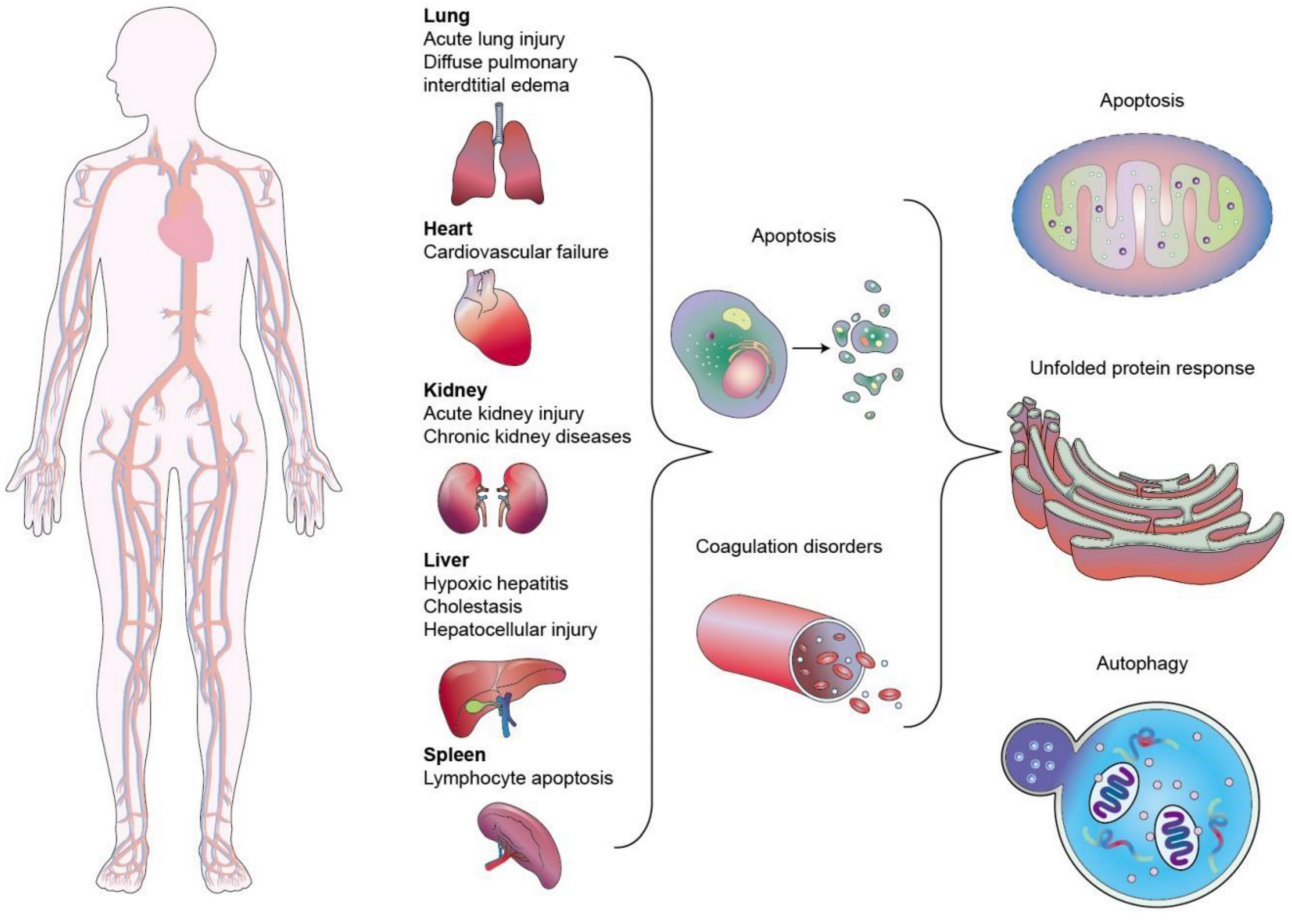
Sepsis in Various Organs. Lungs: In sepsis originating from pneumonia, pathogens invade the lungs, causing inflammation, fluid accumulation, and impaired gas exchange, leading to respiratory distress and acute respiratory distress syndrome (ARDS). Heart: Sepsis-induced myocardial dysfunction can lead to decreased cardiac output and cardiogenic shock, contributing to systemic hypoperfusion and multi-organ failure. Kidneys: Lead to acute kidney injury (AKI) due to decreased blood flow and inflammation, resulting in decreased urine output and accumulation of waste products in the blood. Liver: Leads to hepatic dysfunction and decreased production of clotting factors, resulting in coagulopathy and an increased risk of bleeding. Spleen: In severe spleen sepsis can contribute to multi-organ dysfunction and pose life-threatening complications.

Sepsis is not only a systemic inflammation or immune dysfunction but includes multi-organ failure. At the cellular and molecular levels, sepsis results in the pathological processes of mitochondrial damage, imbalanced inflammatory reaction, abnormal neuroendocrine immune network, immune dysfunction, coagulopathy, endoplasmic reticulum stress, and autophagy (Figure 9). These processes contribute to vasodilation, tissue damage, and peripheral organ injury 72. ALuI is commonly observed as a complication of organ dysfunction in sepsis. ALuI and its severe manifestation - acute respiratory distress syndrome (ARDS) - are acute inflammatory lung diseases characterized by hypoxic respiratory failure and long-term sequelae of lung fibrosis 73. ALuI-associated inflammation increases the permeability of alveolar capillary and lung edema with protein-rich fluid, leading to arterial oxygenation impairment 74. To date, there is no effective pharmacological intervention available for ALuI. The current practice against ALuI involves protective ventilation. ALuI can be a target for local nucleic acid therapy. However, the inefficient delivery of naked nucleic acids hinders its translation into clinical practice. Nanocarriers can potentially resolve this unfavorable feature 75.

### 4.3. Acute heart injury (AHI)

Sepsis-induced AHI refers to a wide spectrum of acute myocardial impairment prompted by sepsis 76. AHI is manifested as myocardial necrosis and cardiac function impairment caused by ventricular remodeling. It triggers a hypoxic microenvironment and extensive myocardial damage. Myocarditis is considered an inflammatory disease that is the leading cause of heart failure and sudden death in young people. With >20% of all cases, myocarditis demonstrates a common cause of cardiac death in young adults 77. Pathogen infection is the most common trigger of AHI and the associated inflammation. Some efforts have been made to develop AHI therapy based on nucleic acids such as miRNAs and mRNAs 78.

### 4.4. Acute kidney injury (AKI)

The kidney plays a principal role in regulating water and electrolyte balance and the excretion of metabolic waste, blood pressure, and acid-base homeostasis. AKI is a syndrome recognized as an abrupt renal function decline. Similar to ALuI, the etiologies of AKI are sepsis, trauma, drug toxicity, and ischemia 79. AKI is identified by intrarenal inflammation, renal tubular injury, endothelial dysfunction, and microcirculation change. It reveals a high mortality rate of about 20% and 50% among hospital and ICU patients, respectively 80. AKI is known to be associated with intrarenal and systemic inflammation. The inflammation in AKI involves the activation of epithelial cells, endothelial cells, resident inflammatory cells, and infiltrating leukocytes. These inflammatory triggers initiate localized and systemic responses through receptors and cells of the innate and adaptive immune systems 81. Current management of AKI mainly focuses on supportive treatments and renal replacement. Emerging evidence supports the feasibility of using nucleic acids and nanocarriers for treating AKI 82. Due to the unique morphology and structure of the kidney, the nanoparticle size greatly affects the efficiency of treatment on AKI. The nanoparticles with the size of <10 nm are easily cleared from the kidney through excretion. In addition, the nanoparticles of 80‒100 nm are suitable for renal retention and prolonged circulation period 83. Cationic nanoparticles are filtered and found in urine with higher amounts compared to their anionic counterparts 84.

### 4.5. Acute liver injury (ALiI)

Sepsis-associated ALiI is one of the manifestations of sepsis-induced multiple organ syndromes. The hepatic inflammatory response, oxidative stress, microcirculation coagulation dysfunction, and bacterial translocation play key roles in the occurrence and development of sepsis-related ALiI 85. Most cases of ALiI have massive hepatocyte necrosis and apoptosis. Hepatocyte necrosis occurs due to ATP depletion causing cellular swelling and cell membrane disintegration 86. A characteristic of ALiI is the upregulation of cytokines by macrophages, including IL-1β and TNF-α, resulting in hyperinflammation and severe hepatic failure 87. Although it has been found that nucleic acids, such as siRNAs and ASOs, can treat liver injury 88, many nucleic acid drugs are underutilized due to their short half-life and side effects. The continuous development of nanosystems has provided new methods for the delivery of nucleic acids to treat liver damage 89.

## 5. The effect of nucleic acid-based nanomedicine for treating sepsis

Hemodynamic stabilization, infection control, and immune response are the three main constituents of sepsis treatment. Antibiotics and anti-inflammatory drugs are the mainstay for controlling infection and inflammation, respectively 90. The problem associated with the current regimen lies in its inability to provide adequate organ perfusion pressure and organ supply, preventing from mitigating organ damage. The side effect of unspecific drug localization is an additional concern 91. Due to the specific regulation of biological function in cells, there has been a growing interest in the application of nucleic acid drugs for the management of sepsis. Despite the advancement in the design of nucleic acids for sepsis treatment, therapeutic outcomes remain unsatisfactory. This can be due to insufficient storage stability, premature nucleic acid release, off-targeting, and inadequate release at the targeted site 92. Such challenges have inspired the introduction of nanomedicine. Following the optimized physicochemical design or surface functionalization with ligands, the nanocarriers allow a controlled and efficient release of nucleic acid drugs to target specific cells or organs, leading to improved therapeutic efficiency and minimized adverse effects. Nowadays, sepsis has become recalcitrant to medical treatment because of multidrug-resistant bacteria. The infection induced by multidrug-resistant microbes demonstrates high morbidity and mortality, causing prolonged hospitalization and elevated healthcare costs 93. The development of nanocarriers for nucleic acids to eradicate the infection and inflammation evoked by drug-resistant pathogens is urgently needed.

For the medical treatment of sepsis, the most common cell for targeting is macrophage 94. Macrophages/monocytes show a capacity for sepsis pathogenesis. In particular, macrophages are resident immunocytes of many organs, exhibiting the mainstay of host defense and inflammation during infection. In sepsis, macrophages are dysfunctional, impairing microbial clearance and increasing susceptibility to secondary infection 95. The bacteria, especially the multidrug-resistant pathogens, evolve an evasion strategy that enables them to survive in macrophages and generates persistent infection in sepsis. The infected macrophages show impermeable membrane penetration and sensitive efflux transporters to antibiotics and anti-inflammatory drugs, resulting in the ineffective eradication of intracellular microbes 96. The activated macrophages in sepsis also play a significant role in immunopathogenesis by secreting a large amount of cytokines/chemokines. The nanoparticles are useful for targeting macrophages to mitigate the severity of sepsis. The nanosystems targeting macrophages depend on the therapeutic aspects to regulate cytokines/chemokines, reprogramming, and programmed death of macrophages 94. In optimizing gene delivery, nanomedicine is critical for achieving extensive application in sepsis treatment. Several studies related to nucleic acid-based nanotherapeutics have been conducted for sepsis mitigation. This intervention of nanomedicine shows an effective relief of sepsis-associated complications and organ dysfunction. Most results of this approach demonstrate superior outcomes compared to naked nucleic acids or conventional therapies.

### 5.1. Bacteremia and Sepsis-induced systemic inflammation

Bacteremia is diagnosed based on the bacterial burden in the bloodstream. Without optimal treatment, bacteremia can progress to sepsis. To mitigate bacteremia-induced sepsis, it is essential to inhibit bacterial growth in circulation. Quercetin is a natural flavonoid that exhibits bacteriostatic and antioxidant activities 97. Sun *et al.* 98 developed quercetin-conjugated Ag nanoparticles stabilized with siRNA for bacteremia treatment. The siRNA chosen in this nanoformulation can silence the wall anchor protein gene in Gram-positive bacteria. The minimum inhibitory concentration (MIC) of the nanosystems and kanamycin against drug-resistant *Bacillus subtilis* is 2.1 and 10.4 μg/ml, respectively. The nanoparticles revealed a high uptake of *B. subtilis* at 100%, according to a fluorescence microscopic analysis. *B. subtilis* was found in blood, lung, heart, kidney, liver, and spleen in a bacteremia model in nude mice. Further, it was found that IV injection of the dual quercetin- and siRNA-loaded nanoparticles decreased *B. subtilis* and inflamed cells in blood and organs. The average mouse weight was increased from 18 to 23 g after the nanoparticle intervention in bacteremia mice for 7 days, approximating the weight of healthy mice (26.1 g). Another case for treating septic shock by siRNA nanoparticles involved the silencing of fat mass and obesity-related protein (FTO) 99. NLRP3 inflammasomes are associated with LPS-induced sepsis 100. LPS is the endotoxin portion of the Gram-negative bacterial cell wall to cause bacteremia-associated hyperinflammation. The siRNA loaded in PEGylated distearoylphosphatidylcholine liposomes is efficiently targeted to macrophages. The transfection of macrophages with the liposomes reduces the FTO mRNA level by four-fold through FoxO1/NF-κB signaling. Intraperitoneal (IP) liposomes inhibit macrophage activation and the associated concentrations of cytokines in circulation, improving the survival rate.

In addition to siRNAs, miRNAs are largely tested for their anti-sepsis activity. Previous investigation 101 has reported that treatment with endothelial progenitor cells increased the circulating level of miR-126, thereby improving the survival of mice receiving cecal ligation and puncture (CLP) sepsis. Jones Buie *et al.* 102 designed deacetylated poly-N-acetyl glucosamine nanoparticles for miRNA-126 delivery to treat sepsis. This kind of biodegradable polymer exhibited a cationic charge that interacted with miRNA. Nanoparticle incubation with NIH 3T3 embryonic fibroblasts increased the levels of miR-126-3p and -5p by 1525- and 3772-fold, respectively. IV treatment with the polymeric nanoparticles improved the survival rate of septic mice at 7 days post-CLP compared to the control (67% versus 25%). The nanoparticles also reduced the serum cytokines and lung/kidney vascular leakage among septic mice. Cancer patients often exhibit an immunosuppression, implying that such a condition may partially defend against sepsis-associated immune overactivation 103. To take advantage of the immunosuppression of tumors without causing harm, Li *et al.* 104 prepared exosomes from tumor cells to appraise their protective effect against sepsis. The exosomes were derived from LPS-stimulated melanoma. Notably, the anti-inflammatory phenotype of RAW264.7 measured by Arg-1 was increased by 2.7-fold after incubation with exosomes. Transcriptome sequencing showed that exosomes majorly functioned through 7 miRNAs (miR-146a-5p, miR-206-3p, miR-466i-5p, miR-615-5p, miR-690, miR-6239, and miR-7651-5p). Exosome mimics prepared by loading the 7 miRNAs in HA-PEI nanoparticles were administered to CLP- or LPS-treated mice via IP. The exosome mimics largely increased the survival rate of mice. The mimics also decreased the levels of IL-6, IL-8, and TNF-α in the serum by 47%, 51%, and 53% in a cynomolgus monkey model, respectively.

mRNAs were effective in treating sepsis by translating them into functional proteins. Hou *et al.* 105 demonstrated that the adoptive transfer of macrophages containing antimicrobial peptides linked to cathepsin B in lysosomes could be applied to the treatment of multidrug-resistant bacteria-induced sepsis. This effect was constructed by transfecting vitamin lipid nanoparticles delivering antimicrobial peptide IB367-cathepsin B mRNA conjugates. The lipid nanoparticles allowed the specific accumulation of IB367-cathepsin B mRNA in macrophage lysosomes, which is the location for bactericidal activity. The lipid nanoparticles with vitamin C were twenty-fold greater than the other vitamins (B3, B7, D, and E) in terms of mRNA delivery into RAW264.7. This nanosystem revealed strong bactericidal activity against drug-resistant *S. aureus* from 33% to 87% at different treatment times (6‒12 h). In the drug-resistant *S. aureus*-induced sepsis mice, the nanosystem with IP or IP+IV significantly eliminated bacterial loading in blood by a 3-log reduction. The survival rate of the vitamin C lipid nanoparticle group (IP+IV) could reach 58% after 30 days. The survival rate of the non-treatment control was 0%. The modification of macrophages with chimeric antigen receptors (CARs) reactivates the phagocytic function against MRSA. *S. aureus* surface protein A (SasA) is an attractive target for identifying MRSA. Tang *et al.* 106 designed an anti-SasA CAR for CAR-macrophage engineering to activate the phagocytic activity of macrophages. In particular, lipid nanoparticles containing SasA-CAR mRNA and caspase-11/caspase-4 (MRSA intracellular evasion target) siRNA were prepared. Caspase-11/caspase-4 is reported to thwart mitochondrial ROS-mediated MRSA killing, enabling MRSA survival in macrophages 107. The lipid nanoparticles delivered mRNA cargo to RAW264.7 with 41% cells expressing SasA-CAR. The nanoparticles killed intracellular MRSA by about five-fold compared to the control after a 24-hour incubation. The IV administration of the mRNA/siRNA-laden nanoparticles in MRSA-infected mice showed a survival percentage of 80%, which was significantly higher than free mRNA and empty nanoparticles.

Yuan *et al.* 108 explored the potential of exosomes from MSCs to mitigate CLP-induced sepsis in mice. In a cell-based study, about 90% of the isolated exosomes were internalized into human umbilical vein endothelial cells (HUVECs). Exosome treatment reduced the expression of IL-6 and IL-8 in the cells. CLP reduced the survival rate to 0% within a 72-hour treatment in mice. The exosomes significantly increased the survival rate to 40%. The nanoparticle intervention also inhibited the expression of IL-6 and IL-8 and the aortic necrosis region. Using the luciferase reporter assay and the database for exploring microRNA-mRNA interaction (starBase), it was suggested that exosomal KCNQ1OT1 played a critical role in suppressing sepsis via the miR-154-3p/RNF19A axis. In addition to exosomes, biomimetic nanoparticles are bioinspired nanocarriers for nucleic acids. Cao *et al.* 109 developed the macrophage membrane-coated zeolite nanoparticles for pDNA delivery to combat sepsis. The antimicrobial gene LL37 was efficiently entrapped into the zeolite imidazolate framework (ZIF-8). Notably, ZIF-8 is reported to enhance pDNA nuclear translocation to ameliorate gene transfection 110. The bone marrow mesenchymal macrophage membrane decoration on nanoparticles allowed targeted delivery of pDNA to RAW264.7 for continuous generation of antimicrobial peptides. The biomimetic nanoparticles showed a 2.5-fold higher LL37 expression than the treatment with a commercial Lipofectamine 2000 transfection kit. The biomimetic nanoparticles cleared blood bacteria with a greater level than the nanoparticles without membrane coating by 1‒2-log in the multidrug-resistant *S. aureus*-induced sepsis in mice. The survival rate of the nanoparticles with and without macrophage membrane was 77% and 65%, respectively. The nucleic acid-based nanotherapeutics for treating bacteremia and systemic sepsis are summarized in Table 2. Information on the nanoparticle size of different nanoformulations is included in this table.

### 5.2. Acute lung injury (ALuI)

When multi-organ damage occurs in sepsis, the lung is highly vulnerable to damage. ALuI and ARDS are two types of lung damage found in sepsis. ARDS can be regarded as the stage where ALuI further deteriorates. The ALuI treatment by drugs should consider facile pulmonary mucus penetration and macrophage retention. The mannose receptor is a specific receptor on the macrophage membrane 111 that can reduce the interaction of mucus 112. Chen *et al.* 113 attempted to promote the mucus transport of chemokine C-C motif receptor 2 (CCR2) siRNA by incorporating mannose and cationic water-soluble pillar arene (CWP) in selenium nanoparticles for treating ALuI. Se nanoparticles are reported to manifest ROS elimination effect against inflammatory injury 114. CWP can act as a stabilizer to synthesize the nanosystem. H_2_O_2_ treatment decreased human bronchial epithelium (HBE) cell survival by 20%. The survival rate of HBE cells reached 99% after siRNA-loaded nanosystem incubation. The Se nanosystem containing mannose and CWP increased mucus permeation capacity by 15-fold in mice. In the LPS-induced ALuI mouse model, the decreased oxygen pressure in arterial blood could be considered a normal level in the nanosystem. The overexpressed ROS, IL-6 and TNF-α concentration in blood was also inhibited under the treatment of the nanosystem. Qian *et al.* 115 attempted to use RNAi for mitigating ALuI by constructing alveolar epithelial cell-targeting PLGA nanoparticles. TNF-α siRNA and alveolar surfactant antibodies were incorporated into the polymeric nanoparticles for RNAi and active targeting, respectively. The nanoparticles significantly decreased the eosinophil number in the lung with intratracheal (IT) instillation of LPS in mice. The RNAi nanocarriers also reduced the expression of IL-1β, IL-6, TNF-α, caspase 3, and B-cell lymphoma-2 in ALuI mice, suggesting a successful outcome in suppressing hyperinflammation.

Zhu *et al.* 116 prepared dopamine-grafted HA nanoparticles coated with poly(β-amino ester) TNF-α siRNA vectors to treat ALuI. This nanocarrier endowed facile mucus penetration through the adhesive nature of dopamine 117. Dopamine-grafted HA is also capable of scavenging ROS to protect the cells from oxidative damage. The polymer-based nanoparticles displayed enhanced mucus penetration *in vitro*. The nanosystem efficiently targeted macrophages due to the CD44 receptor overexpression in the M1 macrophage surface and mediated HA-decorated nanoparticle ingestion. The nasal administration of the nanosystem alleviated cell and protein infiltration in LPS-induced ALuI. The H_2_O_2_ and malondialdehyde were reduced in the injured lung after nanoparticle treatment, indicating the relief of oxidative stress and thereby inhibiting cytokine storms. It is found that cationic polysaccharide nanoparticles provided a high loading to siRNA, and the further coating with pulmonary surfactants (Curosurf) accelerated intracellular siRNA delivery 118,119. Based on this evidence, Merckx *et al.* 120 identified surfactant protein B (SP-B) as the potent pulmonary surfactant to maintain the low surface tension in the lung and enhance intracellular delivery. The cationic dextran nanocomposites were loaded with enhanced green fluorescent protein (eGFP) siRNA. Improved intracellular siRNA delivery was observed by inserting SP-B into the phospholipids prior to nanoparticle coating. 60% eGFP silencing was obtained with 10 nM siRNA in the SP-B-loaded nanoparticles. In the LPS-instilled ALuI mouse model, tracheal aspiration of the SP-B dextran nanoparticles containing TNF-α siRNA reduced TNF-α level to 20% compared to the PBS control.

For better binding to siRNA, Bohr *et al.* 121 prepared dendrimers functionalized with cationic pyrrolidinium and morpholinium surface groups. These secondary amines are considered to have an excellent binding with TNF-α siRNA. The RAW264.7 uptake of pyrrolidinium dendrimers was five- and two-fold higher than naked siRNA and morpholinium dendrimers, respectively. This result could be due to the stronger siRNA complexation and improved protection against enzymatic degradation by pyrrolidinium. In the LPS-induced ALuI murine model, the nasal instillation of the functionalized dendrimers diminished the TNF-α level in the lung by 55% compared to naked siRNA (17%). The recombinant high mobility group box 1A (HMGB1A) peptide is derived from the box A domain of HMGB1. HMGB1A can act as an antagonist of the proinflammatory cytokine HMGB1 122. Oh and Lee 123 combined HMGB1A and S1P (sphingosine-1-phosphate) Lyase siRNA using R3V6 peptide as a carrier to form a nanocomplex. S1P was involved in alveolar integrity and cytokine release. Notably, the downregulation of S1PLyase reduced the activity of LPS-induced mitogen-activated protein kinase (MAPK) signaling 124. The ternary nanocomplex delivered siRNA into the lung epithelial cells more efficiently than lipofectamine and PEI. The administration of the nanocomplex via IT led to a large accumulation in the lung. As a result, it reduced SIPLyase, IL-6, and TNF-α in the bronchial alveolar lavage (BAL) fluid in the LPS-induced ALuI. Minami *et al.* 125 fabricated a nanocomplex of TLR4 siRNA with tetra (piperazino) fullerene epoxide for lung-selective delivery to treat lung damage in sepsis. This nanocomplex formed nano-sized particles (160 nm) in the aqueous solution and agglutinated with plasma proteins to form micro-sized particles (>6 μm). Further, this agglutinate is specifically clogged in pulmonary capillaries to release siRNA into lung cells for RNAi. In the *in vivo* LPS-induced ALuI model, no significant knockdown was observed in all organs except the lung showing a significant 62% silencing. The neutrophil sequestration in the lung of the nanocomplex-treated group was lower than the non-treatment control. The myeloperoxidase (MPO) as the neutrophil marker exhibited a three-fold reduction in the septic mice after nanocomplex intervention.

There are several cases of nanotherapeutic miRNA delivery against ALuI. Some nanoparticles exhibited anti-inflammatory activity to combine with miRNAs for synergizing ALuI therapy. An example is cerium oxide nanoparticles, which are radical scavengers that inhibit the NFκB inflammation pathway for treating coronavirus-associated ALuI 126. Niemiec *et al.* 127 conjugated miRNA-146a with cerium oxide nanoparticles for local delivery to the injured lung. The IT delivery of the metal oxide nanoparticles significantly increased miR-146a concentration in the lung but not the other organs. Bleomycin-damaged lung in mice had increased NO levels from 126 to 169 μM. Nanoparticle treatment lessened NO to the level of baseline control. This treatment prevented ALuI by inhibiting leukocyte recruitment, cytokines (IL-6, IL-8, and TNFα), and collagen deposition. Another approach to synergize ALuI therapy is the combination of miRNAs and anti-inflammatory small-molecule drugs. Li *et al.* 128 developed dendrimer-entrapped gold nanoparticles loaded with miR-155 antagomir and dexamethasone. Gold nanoparticles are useful for reserving the 3D conformation of dendrimers to improve the compression of the genetic materials. The nanosystem downregulated IL-1β, IL-6, and TNF-α in LPS-activated alveolar macrophages based on the effect of gene therapy. Cyclooxygenase (COX)-2 overexpression was also inhibited by the effect of chemotherapy from nanoparticle-associated dexamethasone. Noteworthily, pulmonary edema featured by the increased wet/dry weight ratio is a characteristic of ALuI. The combination of antagomir and dexamethasone completely recovered this value to the normal level relative to healthy control in LPS-induced ALuI. The cytokine level in the BAL fluid of septic mice was lower for the combined nanosystems than for single gene or chemotherapy. Min *et al.* 129 combined miR-194-5p and tetramethylpyrazine for loading into MSNs to treat ALuI. Tetramethylpyrazine is an antioxidant providing anti-inflammatory and anti-apoptotic activities against ALuI 130. The MSNs were functionalized with PEI and PEG for miRNA encapsulation and prolonged half-life. The expression level of miR-194-5p in HUVECs was increased 4‒5 times after the incubation of MSNs for 24 hours. In an animal study, miR-194-5p level was increased 6‒7 times in the lung after the IV administration of MSNs. The combination of both bioactive agents in MSNs increased the survival rate from 30% to 70% at 7 days post-treatment. This effect by MSNs was mediated through the TLR4/NLRP3/caspase 1 axis. Lipid-based nanoparticles were applied to encapsulate miRNAs for lung injury treatment in sepsis. miRNA-146a-loaded lipid nanoparticles consisting of DOPE and DOTAP were coated with mannose to target macrophages for treating septic shock-induced ARDS 131. The mannosylated nanoparticles effectively delivered miRNA-146a into alveolar macrophages and inhibited cytokines IL-8 and macrophage inflammatory protein (*MIP*)-*1α*. ARDS in mice was established by the combination of hemorrhagic shock and mechanical ventilation. The mice treated with IT lipid nanoparticles showed a nearly 10000-fold increase in miRNA-146a level in BAL cells. This increase reduced pulmonary inflammation with the downregulation of IL-6 and chemokine (C-X-C motif) ligand 1 (CXCL1).

Plant-derived exosomes have been proposed as a therapeutic tool for protecting the lungs from ALuI. miRNAs in exosomes from *Rehmanniae radix* are effective in mitigating ALuI 132. The miRNA sequencing assay indicated that miR-7972 exhibited the highest amount among the 50 miRNAs in the exosomes. miRNA-7972 prohibited the production of IL-1β, IL-6, TNF-α, ROS, and NO in LPS-stimulated RAW264.7, facilitating M2 macrophage polarization. miRNA-7972 targeted G protein-coupled receptor 161 (GPR161) to activate the Hedgehog pathway and inhibit *E. coli* biofilm. The *in vivo* study confirmed that exosomes carrying miRNAs were specifically delivered to lung and alveolar macrophages in LPS-treated mice for relieving pulmonary inflammation. Ma *et al.* 133 engineered plant-derived exosomes from *Panax ginseng* by mouse neutrophil membrane coating and investigated their effect on ALuI in sepsis. miRNA-182-5p was transmitted into the engineered exosomes. In the septic mice, the IV administration of miRNA-182-5p-loaded exosomes significantly decreased malondialdehyde and increased superoxide dismutase (SOD) in the serum and lung. The exosomes also restrained the overexpression of IL-1β, IL-6, and TNF-α in ALuI. The same result was observed in the *in vitro* MLE-12 mouse lung type II epithelial cell model. The miRNA-182-5p-loaded exosomes alleviated ALuI via the target regulation of NADPH oxidase 4 (NOX4)/dynamin-related protein 1 (Drp1)/NLRP3 signaling.

SARS-CoV-2 generates lethal pulmonary failure in patients. The virus has spike proteins that bind to human angiotensin-converting enzyme 2 (hACE2) expressed in alveolar cells. The soluble form of hACE2 (hsACE2) can bind to spike protein, preventing viral entry into the cells 134. Kim *et al.* 135 engineered synthetic mRNA to encode hsACE2 for preventing SARS-CoV-2. The lipid nanoparticles composed of distearoylphosphatidylcholine, PEG lipid, and cholesterol were prepared for packaging mRNA. The lipid nanoparticles were internalized into Vero E6 cells to produce hsACE2 protein. This protein effectively suppressed SARS-CoV-2 and its pseudovirus from infecting host cells. The inhaled lipid nanoparticles caused lung transfection in mice and the secretion of mucosal hsACE2 to epithelia - the main site of SARS-CoV-2 pathogenesis. In the case of using pDNA for relieving ALuI, Zhuang *et al.* 136 fabricated dexamethasone-conjugated PEI nanoparticles coated with LA-4 lung epithelial cell membrane to enhance pDNA delivery to the lung. An *in vitro* transfection analysis demonstrated that the biomimetic nanoparticles enhanced the cellular uptake compared with single PEI nanoparticles and the nanoparticles without a cell membrane. Heme oxygenase-1 (HO-1) is an antioxidant enzyme to degrade heme for producing biliverdin and carbon monoxide, which have anti-inflammatory activity. HO-1 pDNA-loaded biomimetic nanoparticles alleviated pulmonary inflammation in the ALuI mouse model after IT administration. This effect was majorly resulted from the synergistic anti-inflammatory activity of dexamethasone and HO-1 pDNA. Notably, pDNA could be employed as a stabilizer of the anti-inflammatory nanocomplex to treat ALuI 137. Glycyrrhizic acid, an anti-inflammatory agent, was loaded into cholesterol-conjugated dendrimers. The flow cytometry verified a more improved L2 lung epithelial cell uptake of glycyrrhizic acid-loaded dendrimers than the blank dendrimers because of the membrane-destabilizing effect of glycyrrhizic acid. The glycyrrhizic acid/pDNA-loaded dendrimers reduced the TNF-α expression in LPS-stimulated RAW264.7. HO-1 pDNA in dendrimers was delivered into the lung via IT in the LPS-treated mice. This treatment increased HO-1 expression in the lung by five-fold. The combined pDNA and glycyrrhizic acid resulted in the reduced TNF-α in BAL fluid by three-fold. The nucleic acid-based nanotherapeutics for treating ALuI are summarized in Table 3.

### 5.3. Acute heart injury (AHI)

AHI and myocarditis are increasingly recognized as symptoms of transient cardiac dysfunction among sepsis patients 138. TLR signaling in response to bacteria triggers intracellular pathways, including the activation of NF-κB and MAPKs. In monocytes and macrophages, TLR activation increases cytokine production to evoke effects on AHI and myocarditis 139. The initial focus on anti-inflammatory therapies in sepsis is important to alleviate AHI. Leuschner *et al.* 140 examined whether *in vivo* delivery of nanoparticle-entrapped siRNA targeting CCR2 reduced inflammation in myocarditis. The lipid-based nanoparticles were prepared based on the compositions of 1,2-distearoyl-sn-glycero-3-phosphocholine, cholesterol, PEG, and cationic lipid C12-200. Twenty-one days after the establishment of myocarditis with subcutaneous (SC) peptide (troponin I peptide VDKVDEERYDVEAKVTKN), the heart contained 1.9×10^5^ monocytes. RNAi of CCR2 via IV reduced the monocyte accumulation to 0.58×10^5^. The cardiac function measured by echocardiography presented the increased left ventricular ejection fraction (LVEF) from 64% to 72% by nanoparticle injection. This finding suggests the recovery of the cardiac function. Meyer *et al.* 141 also used siRNA-loaded lipid nanoparticles to mitigate myocarditis based on the RNAi of colony-stimulating factor 1 (CSF-1). The overexpression of CSF-1 in myocarditis patients controls monocytes originating from hematopoietic stem cells. The lipid nanoparticles were generated as described in Leuschner *et al.* 140. Mouse myocarditis was induced by either SC myosin peptide (SLKLMATLFSTYASAD) or Coxsackievurus B3. CSF-1 expression in the monocytes sorted from the spleen was downregulated by 60% after IV injection of the nanoparticles. In the viral myocarditis model, the flow cytometric assay represented a decrease of CD11b^+^ cells in the heart after the treatment of RNAi nanoparticles (756 versus 273 cells/mg). The CD3^+^ and CD4^+^ T cells were lessened after RNAi (198 versus 95 cells/mg).

Besides siRNAs, myocardial inflammation can be repressed through the treatment of polymer nanoparticle-associated miRNAs. miR-21a-5p antagomir was complexed with PEI to form polymeric nanoparticles 142. Transthoracic echocardiography (TTE) was performed to monitor the LV posterior wall diameter in diastole (LVPWDd) and the LV anterior wall diameter in diastole (LVAWDd) in the myosin peptide-treated mice. Both LVPWDd and LVAWDd increased by 1.2- and 1.4-fold after the induction of myocarditis in mice. The IV treatment of the miR-21a-5p antagomir-PEI complex significantly abolished this increase. In addition, the immunohistochemistry illustrated a remarkable reduction of caspase-1 and NFκB in the heart. The miR-21a-5p expression in the heart was decreased by 21-fold after nanoparticle injection *in vivo*. Another polymeric nanosystem for miRNA delivery to treat AHI involves the use of PEG-PLA nanoparticles with miR-133 143. miR-133 is proven to promote the growth of cardiac myocytes and maintain cardiac homeostasis 144. For testing the effect of nanoparticle-encapsulated miR-133 on cardiac inflammation, AHI was induced in rats by the permanent ligation of the left coronary artery to generate acute myocardial infarction. The nanoparticles were incorporated with an arginine-glycine-aspartic acid tripeptide (RGD) to achieve thrombus targeting. The heart accumulation of the RGD nanoparticles was higher than the nanoparticles without RGD by 1.8-fold after IV administration for 4 hours. The RGD nanoparticles containing miRNA-133 also increased LVEF in the AHI rats. The RGD nanoparticles had lower contents of IL-6, TNF-α, and MPO in the serum compared to naked miRNA and the nanoparticles without RGD.

Adipose-derived stem cell (ADSC)-derived exosomes enriched with miRNAs are beneficial for treating AHI 145. Among several miRNAs in the exosomes, miR-124 plays a fundamental role in inhibiting heart damage in sepsis 146. Luo *et al.* 147 transfected miR-126 agomirs into ADSCs to produce the miR-126-overexpressed exosomes. The exosomes derived from miR-126-overexpressed ADSCs impeded H9c2 myocardial cell damage by reducing IL-1β, IL-6, and TNF-α in a hypoxia environment. The exosomes significantly accelerated microvascular generation and migration in the transwell assay. Acute myocardial infarction in rats could be relieved by exosomes based on the evidence of decreased cytokine expression, apoptosis, and cardiac fibrosis. Human embryonic stem cell (ESC)-derived cardiovascular progenitor cells (CVPCs) are a resource for myocardial repair during heart injury. Wu *et al.* 148 isolated the exosomes secreted from ESC-CVPCs to treat an infarcted heart. It was found that the exosomes enhanced the tube formation and migration of HUVECs. Further, exosomes inhibited an oxygen-glucose deprivation injury in neonatal rat cardiomyocytes through improved cell viability. The exosomes were intramyocardially injected into the mice with cardiac infarction, leading to improved LVEF and vascularization. The RNA sequencing assay presented the abundance of lncRNA metastasis-associated lung adenocarcinoma transcript 1 (MALAT1) in the exosomes.

lncRNA MALAT1 improved tube formation in HUVECs through the targeting of miR-497. The nucleic acid-based nanotherapeutics for treating AHI are summarized in Table 4.

### 5.4. Acute kidney injury (AKI)

Sepsis-associated AKI is a life-threatening complication characterized by an abrupt deterioration of renal function. About 60% of sepsis patients have AKI 149. Microvascular dysfunction, inflammation, and metabolic reprogramming are three mechanisms that play a role in the pathogenesis of sepsis-associated AKI. Similar to ALuI and AHI, the macrophage is the major target of AKI treatment. Histone deacetylase 5 (HDAC5) RNAi in LPS-activated macrophages lessens TNF-α and monocyte chemoattractant protein-1 (MCP-1) 150. In a study, chitosan nanoparticles were constructed to deliver HDAC5 siRNA for treating AKI in a mouse model for oxalate nephropathy 151. The calcium oxalate-activated RAW264.7 treated with the chitosan nanocarriers exhibited a 70% reduction of HDAC5 gene expression compared with the control. The nanoparticle-treated macrophages demonstrated a reduced IL-1β and TNF-α via the regulation of Krüppel-like factor 2 (KLF2) and NLR family pyrin domain containing 3 (NALP3). The same result was found in the *in vivo* AKI murine model. The IV treatment of the siRNA-loaded nanoparticles reduced kidney injury molecule-1 (KIM-1) and neutrophil gelatinase-associated lipocalin (NGAL) by three- and four-fold in the AKI mouse. Another case of AKI treatment by chitosan nanoparticles is RNAi of COX-2 152. The siRNA/chitosan nanoparticles reduced COX-2 at mRNA and protein levels in LPS-stimulated RAW264.7 by 75% and 70%, respectively. The IP injection of the nanocarriers in AKI mice receiving unilateral ureteral obstruction decreased the expression of COX-2, IL-6, and TNF-α to attenuate parenchymal inflammation. The nanoparticles specifically deposited in the inflamed kidney by 2.5-fold compared to the normal kidney.

It is described that the renal expression of C-X-C chemokine receptor 4 (CXCR4) is upregulated in AKI 153. Tang *et al.* 154 modified chitosan nanoparticles with α-cyclam-*p*-toluic acid (CTA) to specifically deliver p53 siRNA to tubule cells in the injured kidney. The CTA-loaded nanoparticles showed 2.1 times greater U2OS cell uptake than the nanoparticles without CTA. The *in vivo* biodistribution in AKI mice with ischemia-perfusion injury demonstrated that IV CTA-loaded nanoparticles had a kidney-to-liver ratio of 1.8, which was higher than the unmodified nanoparticles (0.4). This modified nanoformulation decreased renal apoptosis and macrophage/neutrophil infiltration to improve renal function in AKI mice. This concept was continued by Tang *et al.* 155 to fabricate polymeric CXCR4 antagonist/p53 siRNA polyplexes to treat AKI. Flow cytometry showed that 89% of HK-2 tubule cells ingested the polyplexes, resulting in p53 downregulation by 30% compared to scramble siRNA control. The polyplexes were preferentially accumulated in the injured kidney rather than the healthy kidney by 5.8-fold, protecting the mouse kidney from cisplatin-induced dysfunction.

Several miRNAs in the serum or urine are changed in AKI, making them the potential biomarkers for kidney damage. Aomatsu *et al.* 156 found a significant decrease of miRNA-5100 in the serum of AKI patients by 9.5-fold compared to healthy subjects. The polymer-based nanoparticles composed of PEI were loaded with miRNA-5100 mimic for treating AKI. After the IV injection of the PEI/miRNA nanoparticles in AKI mice with ischemia-perfusion injury, the nanoparticles were mainly located in the tubulointerstitial area according to histological observation. The nanoparticles elevated miR-5100 expression by 2.8-fold in the AKI mice, leading to the decrease of KIM-1 and cleaved caspase-3 in the injured kidney. MSC-derived exosomes are reported to alleviate AKI 157. The exosomal miR-125b-5p derived from MSCs was applicable for suppressing p53 to promote tubular repair 158. The HK-2 cell uptake efficiency of the exosomes was 67% and 36% in the control cells and hypoxia-treated cells, respectively. This high cell internalization of the exosomes could inhibit cell apoptosis via the modulation of B-cell lymphoma 2 (Bcl2) and Bcl2-associated X protein (BAX). An *ex vivo* imaging illustrated the significant delivery of the exosomes to the proximal tubules of ischemic kidneys in mice. The exosome treatment hindered the infiltration of macrophages and CD3^+^ T cells in the kidneys. The tubular epithelial cell arrest in the G2/M phase in ischemia-perfusion was decreased from 16% to 9% after the IV treatment of the exosomes. The proteomic analysis revealed the enrichment of SOD2 in the MSC-derived exosomes. Hou *et al.* 159 prepared lipid nanoparticles to deliver SOD2 mRNA for alleviating AKI. The lipid nanoparticles were mainly found in the mitochondria after incubation with HK-2 cells, leading to the reduced expression of mitochondrial superoxide anion. The intra-adipose capsule delivery of the nanoparticles in the ischemia-reperfusion mice inhibited AKI, as indicated by the decreased level of creatinine and restored tissue integrity. The nucleic acid-based nanotherapeutics for treating AKI are summarized in Table 5.

### 5.5. Acute liver injury (ALiI)

The liver plays a central role in metabolic and immunological homeostasis. These important physiological functions make the liver a major organ for host survival following a severe injury such as sepsis. Hepatic failure - particularly a serious complication in sepsis - directly contributes to disease progression and even death 160. The treatment of anti-inflammatory and antioxidant agents is applicable for restricting the development of ALiI. Fisetin is a natural flavonoid showing anti-inflammatory and antioxidant effects that inhibit macrophage activation 161. Inactive rhomboid protein 2 (iRHOM2) is an inflammation-associated regulator that controls TNF-α production through the crosstalk with TNF-α converting enzyme 162. Xu *et al.* 163 constructed Fe@Au nanoparticles conjugated with fisetin, iRHOM2 siRNA, and TNF-α inhibitor lenalidomide for the synergistic treatment of sepsis-related ALiI. The incubation of the multifunctional metal nanoparticles with listeriolysin-O-activated macrophages significantly reduced the expression of pro-oxidants, including superoxide anion, malondialdehyde, inducible nitric oxide synthase (iNOS), and hydrogen peroxide. The survival rate of *Listeria monocytogenes*-infected mice was 0 within 42 hours after injection. The survival rate could be increased to 60% by the IP treatment of multifunctional nanocarriers. The loss of hepatic function affects coagulation. The prothrombin time of the mice was increased from 12 to 30 seconds after infection - this value could recover to 21 seconds after nanoparticle intervention. Yin *et al.* 164 prepared multifunctional lipid nanoparticles integrated with three active ingredients (i.e., glycyrrhizic acid, polyene phosphatidylcholine, and p65 siRNA) for ALiI management. Polyene phosphatidylcholine is a polyunsaturated fatty acid with versatile bioactivities of anti-inflammation, antioxidation, and immune modulation 165. The multifunctional nanocarriers showed a HeLa cell uptake of 98% according to flow cytometric assay. The IV nanoparticles inhibited the p65 expression in the liver of LPS-treated mice. Furthermore, a significant reduction of IL-1β, IL-6, and COX-2 was found after nanoparticle treatment in LPS-stimulated NCTC1469 cellosaurus cells and LPS-treated mice.

Galactose-conjugated liposomes bearing Fas siRNA were designed as a hepatocyte-specific delivery system for the treatment of ALiI 166. Galactose-guided nanoparticles target hepatocytes specifically via the asialoglycoprotein receptor. The IV injection of galactose-conjugated liposomes prolonged the circulation time of siRNA.

The fluorescence from the Cy3-labeled siRNA was detectable in the liver after a 48-hour administration. The Fas gene silence effect in the liver was 90% after liposomal administration. In the concanavalin A-induced ALiI mouse model, the liposomes significantly inhibited hepatic inflammation and apoptosis. Ding *et al.* 167 synthesized ionizable lipids through Michael's addition reaction using alkyl-acrylate and amines with spermine as the amine-head moiety. The synthesized lipid was mixed with dioleoylphosphatidylethanolamine, cholesterol, and PEG to form lipid nanoparticles for delivering IL-1β siRNA against ALiI. This ionizable lipid nanosystem facilitated RAW264.7 uptake through multiple pathways, including micropinocytosis, clathrin-, and caveolae-mediated endocytosis. A 70% IL-1β silencing in primary macrophages was achieved by the ionizable lipid nanoparticles. The IV treatment of the nanocarriers downregulated the IL-1β expression in the liver and spleen of LPS-treated mice by 65% and 42%, respectively. Noteworthily, the infiltrated immune cells and disarranged hepatocytes in the injured liver were lessened by the nanomedicine. Ionizable lipid nanoparticles were also prepared by synthesizing the ionizable lipids through a one-step ring-opening reaction between 1,2-epoxytetradecane and four amine-rich molecules 168. The nanosystem loaded with IL-1β siRNA exhibited efficient internalization and endosomal escape in RAW264.7. The *in vivo* imaging system (IVIS) illustrated that the nanosystem is mainly deposited in the liver to about 80% of all organs. There were >70% of the immune cells labeled by the fluorescent nanoparticles in the liver. The overexpressed IL-1β in the liver and spleen of LPS-treated mice was inhibited by 60% after nanoparticle treatment, mitigating hepatic tissue damage.

Cationic helical polypeptide PPABLG with a rigid amphiphilic structure can be a cell-penetrating peptide that destabilizes cell membranes to facilitate cellular uptake. He *et al.* 169 prepared polypeptide nanoparticles based on PPABLG for the efficient delivery of TNF-α siRNA to the liver. This nanocarrier revealed a greater RAW264.7 uptake of >80% compared to lipofectamine (10%). The TNF-α production in LPS-activated RAW264.7 was reduced by 90% after nanoparticle treatment. In the *in vivo* LPS-treated murine model, the IV administration of the polypeptide nanoparticles decreased serum TNF-α by 94%. The TNF-α level was also downregulated in the macrophage-enriched organs, including the liver, spleen, and lung. The survival rate within 10 days was 50% for the nanoparticle group, which was higher than the commercial gene delivery vector (20%). Most of the gene-based nanocarriers are administered via injection route. Nevertheless, oral delivery is a convenient route with less invasiveness. However, a major concern of the orally administered nucleic acid nanoparticles is the need to penetrate across the gastrointestinal membrane and the avoidance of nucleic acid degradation in the harsh environment of the gastrointestinal tract. Hence, He *et al.* 170 developed mannose-modified trimethyl chitosan-cysteine conjugate nanoparticles for the oral delivery of TNF-α siRNA. This nanoformulation could be activated at a suitable time and location to overcome the extracellular and intracellular barriers. This type of nanosystem efficiently maintained siRNA integrity in a physiological environment, increased siRNA transport across the intestinal epithelium, enhanced siRNA ingestion into macrophages, and promoted cytoplasmic siRNA release. A 70% TNF-α silencing in RAW264.7 was obtained by nanoparticle incubation. This effect was 200-fold higher than that of lipofectamine. The oral gavage of the nanocarriers abolished LPS-induced TNF-α overexpression in mice, with a 90% reduction at siRNA dose of 15 nM/kg. Further, the nanosystem protected the animals from ALiI from inflammation-evoked liver damage and lethality. He *et al.* 171 further prepared an optimized mannose-modified trimethyl chitosan-cysteine conjugate nanoparticles using different anionic crosslinkers, including tripolyphosphate (TPP), HA, and Eudragit S100. The *in vitro* TNF-α silencing in RAW364.7 demonstrated a prolonged and prompt RNAi by the treatment of both nanoformulations using TPP:HA:ES=1:1:1 and TPP alone. In the LPS-treated ALiI murine model, the serum TNF-α knockdown by the nanoparticles using TPP alone was twice higher than those using three anionic crosslinkers. The nanoparticles using TPP alone rapidly released siRNA in the cytosol and mediated 24-hour gene silencing in macrophages.

Rizvi *et al.* 172 employed mRNA-based nanotherapeutics to accelerate liver regeneration in the acetaminophen-induced ALiI. Hepatocyte growth factor (HGF) and epidermal growth factor (EGF) mRNAs were loaded by lipid nanoparticles composed of ionizable cationic lipids, phosphatidylcholines, cholesterol, and PEG. After the IV administration to the mice, hepatocytes were the predominant cells targeted by the lipid nanoparticles, together with endothelial cells and Kupffer cells in the liver. The protein production by the IV mRNA-loaded nanocarriers could last about 3 days. The HGF mRNA-loaded nanocarriers effectively accelerated hepatocyte proliferation according to the absence of terminal deoxynucleotidyl transferase dUTP nick end labeling (TUNEL^+^) cells in the hepatic histology. The nucleic acid-based nanotherapeutics for treating ALiI are summarized in Table 6.

## 6. Conclusions and future perspectives

Gene therapy holds immense potential for clinical therapy due to its role as a vital regulator of cell behavior in pathological conditions. The nucleic acid drugs can suppress or promote the expression of several genes to realize the aim of treating or preventing bacterial infection and inflammation. Sepsis and its related organ injury can be the target disease for efficient nucleic acid therapy. The powerful and safe delivery of nucleic acids, such as siRNAs, miRNAs, mRNAs, ASOs, and pDNAs, is of paramount importance for their exploitation in clinical application, considering that naked nucleic acids are susceptible to biodegradation. In addition, the off-target effect of the naked nucleic acid drugs minimizes the therapeutic effect. Although a chemical modification of the nucleic acids can improve the bioavailability and targeting to specific cells or tissues, this strategy is still unsatisfactory for prolonging the half-life. Moreover, many nucleic acids are not suitable for chemical modification. As the vehicles for nucleic acids, nanoparticles can overcome delivery challenges. The nanostructures possess unique physicochemical and biological features that can be of interest in the engineering of drug delivery systems for treating diseases. The possibility of designing nanocarriers with specific properties - such as size, surface charge, shape, elasticity, and functionality - allows the application of nucleic acids in biomedical and therapeutic areas. In the application of sepsis treatment, nanoparticle-encapsulated nucleic acids enhance the targeting efficacy through the change of nanostructure materials and physicochemical characteristics. Consequently, the anti-sepsis nanoparticles can deliver nucleic acids to different organs based on the unique features of organs or tissues.

Most nucleic acid nanomedicines for treating sepsis have only been tested in cell-based and animal-based studies. There have been very few clinical trials to date. Although the disease-like model in animals is considered a promising approach to simulating human diseases, there remain differences between the pathogenesis of experimental and human diseases. Whether the nucleic acid-based nanotherapeutics evaluated by the animal model are still successful in clinical sepsis conditions needs further verification. For future anti-sepsis therapy perspectives in using nucleic acid-loaded nanocarriers, some limitations should be resolved to elevate the clinical application. Most research on the nanomedical application of sepsis has designed nanostructures at a lab scale. Therefore, for future clinical applications, manufacturing and scalability are important factors to consider in nucleic acid nanoparticles. However, the current challenge for the scale-up of the nanoparticles lies in the high cost of the materials, including nucleic acids, functional components, and manufacturing devices/machines. Complex delivery systems also raise the problem of reproducibility. Cost-effectiveness is the substantial purpose of improving the current drawbacks of nanocarrier application. Nanocarriers are largely transported to the liver after intravenous injection since foreign materials are easily tracked toward this organ. Many nanoparticles for nucleic acid delivery are suitable for treating liver-related diseases. However, it is challenging to efficiently deliver nucleic acids to other injured organs or tissues.

To avoid the preferential hepatic uptake of nanocarriers for treating sepsis, physicochemical modification of nanoparticles and new administration routes should be considered. Several nucleic acid types have been proven useful for treating septic inflammation. However, it is impossible to design a one-size-fits-all nanosystem for all the nucleic acids to target specific cells or organs. The use of nucleic acid as the drugs is also a challenge to treat sepsis. Sepsis is a highly heterogeneous syndrome with diverse etiologies, leading to the difficulty to identify universal therapeutic targeting. The inflammatory state in sepsis can induce an immune reaction against foreign nucleic acids. That is why there are no approved nucleic acid drugs for sepsis management. Thus the further development of novel nucleic acids is important as well for combining with nanocarriers to achieve efficient delivery and therapy. Nanodelivery systems will be of continuous interest in improving nucleic acid delivery and therapy. Moreover, the re-evaluation and progressive design of the nanoformulation for sepsis treatment are necessary for optimizing therapeutic efficacy.

## Figures and Tables

**Table 1:** Nucleic acid drugs approved by USFDA or European Medicines Agency

Year of approval	Name	Type	Target	Indication	Administration
1998	Fomivirsen	ASO	CMV IE2	Cytomegalovirus ritinitis	Intraocular
2004	Pegaptanib	aptamer	VEGF	Neovascular age-related macular degeneration	Intraocular
2013	Mipomersen	ASO	ApoB-100	Homozygous familial hypercholesterolemia	Subcutaneous
2016	Eteplirsen	SSO	Exon 51	Duchenne muscular dystrophy	Intravenous
2016	Nusinersen	SSO	Exon 7	Spinal muscular atrophy	Intrathecal
2018	Inotersen	ASO	TTR	Hereditary transthyretin mediated amyloidosis	Subcutaneous
2018	Patisiran	siRNA	TTR	Hereditary transthyretin mediated amyloidosis	Intravenous
2019	Golodirsen	SSO	Exon 53	Duchenne muscular dystrophy	Intravenous
2019	Volanesorsen	ASO	ApoC3	Familial chylomicronemia syndrome	Subcutaneous
2019	Givosiran	siRNA	ALAS1	Acute hepatic porphyria	Subcutaneous
2020	Viltolarsen	SSO	Exon 53	Duchenne muscular dystrophy	Intravenous
2020	Lumasiran	siRNA	Hydroxyacidoxidase 1	Primary hyperoxaluria type 1	Subcutaneous
2020	Inclisiran	siRNA	PCSK9	Familial hypercholesterolemia	Subcutaneous
2020	Tozinameran	mRNA	SARS-CoV-2	COVID-19 vaccine	Intramuscular
2020	Elasomeran	mRNA	SARS-CoV-2	COVID-19 vaccine	Intramuscular
2021	Casimersen	SSO	Exon 45	Duchenne musculardystrophy	Intravenous
2022	Vutrisiran	siRNA	TTR	Hereditary transthyretin-mediated amyloid polyneuropathy	Subcutaneous
2023	Tofersen	ASO	mRNA for SOD1	Amyotrophic lateral sclerosis	Intrathecal
2023	Eplontersen	ASO	TTR	Transthyretin-mediated amyloidosis	Subcutaneous
2023	Nedosiran	siRNA	Lactate dehydrogenase A	Primary hyperoxaluria	Subcutaneous
2023	Avacincaptad pegol	aptamer	Complement factor C5	Age-related macular degeneration	Intravitreal

ALAS1, 5'-aminolevulinate synthase 1; ApoC3, apolipoprotein C-III; ASO, antisense oligonucleotide; CMV IE2, cytomegalovirus IE2; PCSK9, proprotein convertase subtilisin-like kexin type 9; SARS-CoV-2, severe acute respiratory syndrome coronavirus 2; SOD1, superoxide dismutase 1; SSO, splice switching oligonucleotide; TTR, transthyretin; VEGF, vascular endothelial growth factor.

**Table 2:** The nucleic acid-based nanotherapeutics for treating sepsis-induced systemic inflammation and bacteremia

Nucleic acid type	Nanoparticle type	Size (nm)	Cell model	Animal model	Delivery route	Reference
siRNA targeting wall anchor protein	Silver	About 50	*B. subtilis*	Bacteremia mouse model	IV	Sun *et al.* 98
siRNA targeting FTO	Liposomes	60‒120	Mouse macrophages	LPS-induced sepsis in mice	IP	Luo *et al.* 99
miR-126-3p and -5p	Polymer	204	NIH 3T3 embryonic fibroblasts	CLP-induced sepsis in mice	IV	Jones Buie *et al.* 102
miR-146a-5p, miR-206-3p, miR-466i-5p, miR-615-5p, miR-690, miR-6239, and miR-7651-5p	Exosomes	138	RAW264.7 macrophages	LPS-induced sepsis in mice and cynomolgus monkey	IP	Li *et al.* 104
Cathepsin B mRNA	Lipid	127‒174	RAW264.7 macrophages	Drug-resistant *S. aureus*-induced sepsis in mice	IP and IV	Hou *et al.* 105
CAR mRNA and caspase-11/caspase-4 siRNA	Lipid	About 150	RAW264.7 macrophages	MRSA-induced sepsis in mice	IV	Tang *et al.* 106
lncRNA KCNQ1OT1	Exosomes	About 100	*HUVECs*	CLP-induced sepsis in mice	IV	Yuan *et al.* 108
LL37 pDNA	Macrophage- coated biomimetic nanoparticles	155	RAW264.7 macrophages	Drug-resistant *S. aureus*-induced sepsis in mice	IV	Cao *et al.* 109

CAR, chimeric antigen receptor; CLP, cecal ligation and puncture; FTO, fat mass and obesity-related protein; HUVECs, human umbilical vein endothelial cells; IP, intraperitoneal; IV, intravenous; LPS, lipopolysaccharide; MRSA, methicillin-resistant *S. aureus*.

**Table 3:** The nucleic acid-based nanotherapeutics for treating sepsis-induced acute lung injury (ALuI)

Nucleic acid type	Nanoparticle type	Size (nm)	Cell model	Animal model	Delivery route	Reference
siRNA targeting CCR2	Selenium	160‒220	HBE cells	LPS-induced ALuI in mice	IT	Chen *et al.* 113
siRNA targeting TNF-α	Polymer	About 250	None	LPS-induced ALuI in mice	IV	Qian *et al.* 115
siRNA targeting TNF-α	Polymer	About 150	RAW264.7 macrophages	LPS-induced ALuI in mice	Nasal	Zhu *et al.* 116
siRNA targeting eGFP and TNF-α	Polymer	<200	H1299 lung epithelial cells	LPS-induced ALuI in mice	IT	Merckx *et al.* 120
siRNA targeting TNF-α	Dendrimers	120‒190	RAW264.7 macrophages	LPS-induced ALuI in mice	Nasal	Bohr *et al.* 121
siRNA targeting S1PLyase	Peptide	191	RAW264.7 macrophages and LA-4 lung epithelial cells	LPS-induced ALuI in mice	IT	Oh and Lee 123
siRNA targeting TLR4	Fullerene	160	None	LPS-induced ALuI in mice	IV	Minami *et al.* 125
miRNA-146a	Cerium oxide	About 5	Mouse fibroblasts	Bleomycin- induced ALuI in mice	IT	Niemiec *et al.* 127
miRNA-144 antagomir	Gold	175‒230	Mouse alveolar macrophages	LPS-induced ALuI in mice	Nasal	Li *et al.* 128
miR-194-5p	MSNs	100‒200	HUVECs	LPS-induced ALuI in mice	IV	Min *et al.* 129
miRNA-146a	Lipid	219	Human alveolar macrophages	Hemorrhagic shock-induced ARDS in mice	IT	Fei *et al.* 131
miR-7972	Exosomes	118	RAW264.7 macrophages	LPS-induced ALuI in mice	IV	Qiu *et al.* 132
miR-182-5p	Exosomes	130	MLE-12 mouse lung type II epithelial cells	LPS-induced ALuI in mice	IV	Ma *et al.* 133
hsACE2 mRNA	Lipid	About 80	Vero E6	LPS-induced ALuI in mice	IT and IH	Kim *et al.* 135
HO-1 pDNA	Biomimetic	About 80	LA-4 and L2 lung epithelial cells	LPS-induced ALuI in mice	IT	Zhuang *et al.* 136
HO-1 pDNA	Dendrimers	107	RAW264.7 macrophages and L2 lung epithelial cells	LPS-induced ALuI in mice	IT	Choi *et al.* 137

ARDS, acute respiratory distress syndrome; CCR2, C-C motif receptor 2; eGFP, enhanced green fluorescent protein; HBE, human bronchial epithelium; hsACE2, the soluble form of human angiotensin-converting enzyme 2; HO-1, heme oxygenase-1; HUVECs, human umbilical vein endothelial cells; IH, inhalation; IT, intratracheal; IV, intravenous; MSNs, mesoporous silica nanoparticles; S1P, sphingosine-1-phosphate; TLR4, toll-like receptor 4; TNF-α, tumor necrosis factor-α.

**Table 4:** The nucleic acid-based nanotherapeutics for treating sepsis-induced acute heart injury (AHI)

Nucleic acid type	Nanoparticle type	Size (nm)	Cell model	Animal model	Delivery route	Reference
siRNA targeting CCR2	Lipid	60‒80	None	Pathogenic peptide-induced myocarditis in mice	IV	Leuschner *et al.* 140
siRNA targeting CSF-1	Lipid	Unknown	NIH-3T3 cells	Pathogenic peptide- and Coxsackievurus B3-induced myocarditis in mice	IV	Meyer *et al.* 141
miRNA-21a-5p antagomir	Polymer	Unknown	None	Pathogenic peptide-induced myocarditis in mice	IV	Mirna *et al.* 142
miRNA-133	Polymer	138	None	Ligation of left coronary artery in rats	IV	Sun *et al.* 143
miRNA-126	Exosomes	50‒100	H9c2 myocardial cells	Ligation of left coronary artery in rats	IV	Luo *et al.* 147
lncRNA MALAT1	Exosomes	118	HUVECs	Ligation of left coronary artery in mice	Intramyocardial	Wu *et al.* 148

CCR2, C-C motif receptor 2; CSF-1, colony stimulating factor 1; HUVECs, human umbilical vein endothelial cells; IV, intravenous; MALAT1, metastasis associated lung adenocarcinoma transcript 1.

**Table 5:** The nucleic acid-based nanotherapeutics for treating sepsis-induced acute kidney injury (AKI)

Nucleic acid type	Nanoparticle type	Size (nm)	Cell model	Animal model	Delivery route	Reference
siRNA targeting HDAC5	Polymer	264	RAW264.7 macrophages	Oxalate-induced AKI model in mice	IV	Sharma *et al.* 151
siRNA targeting COX-2	Polymer	Unknown	RAW264.7 macrophages	Unilateral ureteral obstruction in mice	IP	Yang *et al.* 152
siRNA targeting p53	Polymer	About 130	HK-2 proximal tubule cells and U2OS cells	Ischemia-perfusion injury in mice	IV	Tang *et al.* 154
siRNA targeting p53	Polymer	107‒300	HK-2 proximal tubule cells	Cisplatin- induced AKI model in mice	IV	Tang *et al.* 155
miRNA-5100	Polymer	Unknown	None	Ischemia-perfusion injury in mice	IV	Aomatsu *et al.* 156
miRNA-125b-5p	Exosome	134	HK-2 proximal tubule cells	Ischemia-perfusion injury in mice	IV	Cao *et al.* 158
SOD2 mRNA	Lipid	About 100 nm	HK-2 proximal tubule cells	Ischemia-perfusion injury in mice	Intra-adipose capsule	Hou* et al.* 159

COX-2, cyclooxygenase-2; HDAC5, histone deacetylase 5; IP, intraperitoneal; IV, intravenous; SOD2, superoxide dismutase 2.

**Table 6:** The nucleic acid-based nanotherapeutics for treating sepsis-induced acute liver injury (ALiI)

Nucleic acid type	Nanoparticle type	Size (nm)	Cell model	Animal model	Delivery route	Reference
siRNA targeting iRHOM2	Iron and gold	15	THP-1 cells and PBMC cells	*Listeria monocytogenes*-infected mice	IP	Xu *et al.* 163
siRNA targeting p65	Lipid	133	HeLa and NCTC1469 cellosaurus cells	LPS-induced ALiI in mice	IV	Yin *et al.* 164
siRNA targeting Fas	Liposomes	116	None	concanavalin A-induced ALiI IN mIce	IV	Jiang *et al.* 166
siRNA targeting IL-1β	Lipid	About 160	RAW264.7 and primary peritoneal macrophages	LPS-induced ALiI in mice	IV	Ding *et al.* 167
siRNA targeting IL-1β	Lipid	130‒170 nm	RAW264.7 and primary peritoneal macrophages	LPS-induced ALiI in mice	IV	Zhang *et al.* 168
siRNA targeting TNF-α	Polypeptide	About 100 nm	RAW264.7 macrophages	LPS-induced ALiI in mice	IV	He *et al.* 169
siRNA targeting TNF-α	Polymer	147 nm	RAW264.7 and primary peritoneal macrophages and Caco-2 cells	LPS-induced ALiI in mice	Oral	He *et al.* 170
siRNA targeting TNF-α	Polymer	120‒225	RAW264.7 macrophages	LPS-induced ALiI in mice	Oral	He *et al.* 171
HGF and EGF mRNAs	Lipid	About 80	None	Acetaminophen- induced ALiI in mice	IV	Rizvi *et al.* 172

EGF, epidermal growth factor; HGF, hepatocyte growth factor; IL-1β, interleukin-1β; IP, intraperitoneal; IV, intravenous; iRHOM2, inactive rhomboid protein 2; PBMC, human peripheral blood mononuclear cell; TNF-α, tumor necrosis factor-α
